# Transcriptomic profiling of gene expression and RNA processing during *Leishmania major* differentiation

**DOI:** 10.1093/nar/gkv656

**Published:** 2015-07-06

**Authors:** Laura A. L. Dillon, Kwame Okrah, V. Keith Hughitt, Rahul Suresh, Yuan Li, Maria Cecilia Fernandes, A. Trey Belew, Hector Corrada Bravo, David M. Mosser, Najib M. El-Sayed

**Affiliations:** 1 Department of Cell Biology and Molecular Genetics, 3128 Bioscience Research Building, University of Maryland, College Park, MD 20742, USA; 2 Center for Bioinformatics and Computational Biology, University of Maryland, College Park, MD 20742, USA; 3 Department of Mathematics, University of Maryland, College Park, MD 20742, USA; 4 Department of Computer Science, University of Maryland, College Park, MD 20742, USA

## Abstract

Protozoan parasites of the genus *Leishmania* are the etiological agents of leishmaniasis, a group of diseases with a worldwide incidence of 0.9–1.6 million cases per year. We used RNA-seq to conduct a high-resolution transcriptomic analysis of the global changes in gene expression and RNA processing events that occur as *L. major* transforms from non-infective procyclic promastigotes to infective metacyclic promastigotes. Careful statistical analysis across multiple biological replicates and the removal of batch effects provided a high quality framework for comprehensively analyzing differential gene expression and transcriptome remodeling in this pathogen as it acquires its infectivity. We also identified precise 5′ and 3′ UTR boundaries for a majority of *Leishmania* genes and detected widespread alternative *trans*-splicing and polyadenylation. An investigation of possible correlations between stage-specific preferential *trans-*splicing or polyadenylation sites and differentially expressed genes revealed a lack of systematic association, establishing that differences in expression levels cannot be attributed to stage-regulated alternative RNA processing. Our findings build on and improve existing expression datasets and provide a substantially more detailed view of *L. major* biology that will inform the field and potentially provide a stronger basis for drug discovery and vaccine development efforts.

## INTRODUCTION

Obligate intracellular protozoan parasites of the genus *Leishmania* are the causative agents of leishmaniasis, a group of diseases with a worldwide incidence of 0.9–1.6 million cases per year. The disease can vary in severity from self-healing skin lesions to disfiguring mucosal manifestations to fatal visceral disease (1). The parasite's life cycle is divided between its mammalian host, where it resides inside of host macrophages, and its insect vector, the phlebotomine sand fly. When responding to changes in the environment as it moves through its life cycle, such as upon leaving the sand fly vector and infecting host cells, the parasite must adapt to its new surroundings. While some of these adaptations can be seen as changes in morphology (size, shape, position of organelles) and variations in cell surface component (2–4), less is known about the global changes that take place at the transcriptomic level.

Unlike most other eukaryotes, *Leishmania* and other trypanosomatids, including *Trypanosoma brucei* and *Trypanosoma cruzi*, do not regulate the expression levels of individual genes by the differential recruitment of RNA polymerase II influenced by cellular transcription factors. Rather, their genes are arranged as polycistronic clusters of tens to hundreds of functionally unrelated genes which are transcribed at roughly the same rate across the genome (5–7). The *trans*-splicing of a capped 39-nucleotide (nt) spliced leader (SL) mini-exon sequence to the 5′ end of each nuclear mRNA and the polyadenylation of the 3′ end are used to separate each polycistronic pre-mRNA transcript into its component mature mRNAs (8). A number of other organisms, ranging from dinoflagellates to nematodes to chordates, exhibit evidence of the *trans*-splicing of an SL sequence to at least a subset of their genes (9–13).

In trypanosomatids, transcription initiation sites occur at divergent ‘strand switch regions’ where polycistronic units originate in opposite directions on opposing DNA strands (7,14,15). *Trans*-splicing and polyadenylation events are coupled temporally and spatially such that the SL acceptor site of the downstream gene determines the location of the polyadenylation site of the upstream gene and both modification events occur simultaneously during post-transcriptional processing (16–18). SL acceptor sites contain a consensus AG dinucleotide that is preceded by polypyrimidine-rich sequence and a G nucleotide excluded from the −3 position (16,17,19). Polyadenylation sites do not appear to contain a specific signal sequence and have been reported to occur about 500–600 nt upstream of the coupled *trans*-splicing acceptor site (16).

Steady-state mRNA levels for individual genes are largely dependent on gene copy number and the rate of mRNA degradation, with mRNA deadenylation preceding degradation for most mRNAs. Sequence motifs contained in the 3′ UTRs greatly influence mRNA stability and the recruitment of the cellular degradation machinery (20–30). Since kinetoplastids lack introns (with very few exceptions), they do not control gene expression by alternative *cis*-splicing (6,31). Gene expression is thus predominantly controlled, not at the transcriptional level through the developmental regulation of RNA polymerase II activity, but by gene copy number, post-transcriptional mRNA processing, rates of mRNA degradation and translational efficiency (see (32) for review).

The genome sequences of *Leishmania major, T. brucei*, and *T. cruzi* were completed in 2005 (6,33,34), yet much remains unknown about the boundaries of individual genes and the mechanisms directing the expression levels of individual genes. Most previous studies examining *Leishmania* gene expression have relied on SAGE tags or on microarrays (35–44). While very informative, microarray-based approaches have several inherent limitations such as hybridization and cross-hybridization artifacts, the restriction on genes interrogated to probes included on the array (inhibiting the identification of previously unannotated genes), dye-based detection issues, the need for large amounts of input RNA and the inability to detect 5′ and 3′ UTRs boundaries. Furthermore, comparison of results between studies has been hindered by differences in the developmental stages studied and the probes included on the microarrays. These limitations likely resulted in the identification of an incomplete list of genes that are up- or downregulated in the various life cycle stages. RNA-seq, which enables a precise and sensitive measurement of mRNA transcript abundance, has begun to be applied to this problem (45), and additional, comprehensive, well-replicated studies examining gene expression across multiple conditions are needed to more fully understand both the gene expression signatures of individual developmental stages and the changes that take place as the parasite progresses through its life cycle.

In this study, we performed transcriptome profiling using RNA-seq to identify global changes in gene expression that occur as *L. major* undergoes metacyclogenesis from the proliferative, non-infective procyclic promastigote form to the non-dividing, infective metacyclic promastigote form, a developmental progression that is well mimicked *in vitro* using reliable axenic cultivation methods (46). Differential gene expression analysis enabled us to distinguish between the procyclic promastigote and metacyclic promastigote developmental forms and shed light on how the parasite alters gene expression as it achieves infectivity. We precisely identified the 5′ and 3′ UTR boundaries for a majority of *Leishmania* genes and detected widespread alternative *trans*-splicing and polyadenylation. A paired-end mRNA sequencing approach was used to allow high confidence read mapping and transcript assembly. Collection of data from multiple biological replicates, careful statistical analysis of variation and removal of batch effects provided us with a unique ability to detect biological differences between the two developmental stages with enhanced confidence and sensitivity. The resources generated by this work build on and improve existing expression datasets and gene structure annotations and provide a substantially more detailed interpretation of *L. major* biology that will inform the field and potentially provide additional data for drug discovery and vaccine development efforts.

## MATERIALS AND METHODS

### *Leishmania* culture

*Leishmania major* (clone V1, MHOM/IL/80/Friedlin) was isolated after passage through BALB/c mice. Promastigotes were grown in 50% M199 39% Schneider medium along with 10% Fetal Bovine Serum (FBS) and 1% of Penicillin/streptomycin at 25°C. *L. major* promastigotes were not split for more than five passages to maintain virulence of the cultures. Enrichment for metacyclic promastigotes from stationary phase cultures was done by Ficoll density gradient centrifugation (47) and by negative selection with peanut agglutinin (PNA) (48). Six biological replicates, each originating from a separate growth and obtained on different days, were collected for procyclic promastigotes and Ficoll-enriched metacyclic promastigotes. For three of the growths, metacyclic promastigotes were also collected following PNA selection.

### RNA isolation and cDNA library preparation

Total RNA was isolated using the Trizol^®^ reagent (Invitrogen, CA, USA), treated with DNase and purified using the Qiagen RNeasy mini kit. RNA integrity was assessed using an Agilent 2100 bioanalyzer. Poly(A)^+^-enriched cDNA libraries were generated using the Illumina TruSeq Sample Preparation kit (San Diego, CA, USA) and checked for quality and quantity using the bioanalyzer and qPCR (KAPA Biosystems).

### RNA-seq data generation, pre-processing and quality trimming

Paired end reads (100 bp) were obtained from the Illumina HiSeq 1500 platform. Trimmomatic (49) was used to remove any remaining Illumina adapter sequences from reads and to trim bases off the start or the end of a read when the quality score fell below a threshold of 20. Sequence quality metrics were assessed using FastQC (http://www.bioinformatics.babraham.ac.uk/projects/fastqc/).

### Mapping cDNA fragments to the reference genome, abundance estimation and data normalization

Reads were aligned to the *L. major* genome (v. 6.0) obtained from the TriTrypDB database (www.tritrypdb.org) using TopHat (v 2.0.10) (50). Two mismatches per read were allowed and reads were allowed to map only to a single locus. The abundance of reads mapping to each gene feature in the TriTrypDB *L. major* annotation (v 6.0) was determined using HTSeq (51). The resulting count table was restricted to protein-coding genes. A count table was also generated that included the above features along with a set of novel open reading frames (ORFs) of at least 90 nt in length. These novel ORFs were identified by manual annotation of translational evidence from a ribosome profiling study of *L. major* procyclic promastigote samples. Their coordinates are provided in Dataset S1 and have been transmitted to TriTrypDB for the assignment of unique gene identifiers.

### Data quality assessment by statistical sample clustering and visualization

Multiple approaches were used to evaluate replicates and to visualize sample-sample distances. Those included Pearson correlation, median pairwise correlation (MPC) analysis, box plots, principal component analysis (PCA) and Euclidean distances-based hierarchical clustering. Samples that did not pass the following quality assessment procedure were excluded from downstream analyses. For each sample, the MPC to all other samples in the dataset was computed (Supplementary Figure S1C). A standard outlier identification method (52) was applied to remove samples that have low correlation with the other samples. Samples were removed if their median MPC was less than Q1 (MPC)–1.5 IQR (MPC) where Q1 (MPC) and IQR (MPC) represent the first quartile and inter-quartile range of the MPC across all samples, respectively. Two samples from a single sequencing batch (batch A) were removed as a result.

### Differential expression analysis

Non-expressed and weakly expressed genes, defined as having <1 read per million in n of the samples, where n is the size of the smallest group of replicates (here *n* = 5), were removed prior to differential expression (DE) analysis (53). A quantile normalization scheme was applied to all samples (54). Following log2 transformation of the data, limma (a Bioconductor package) was used to conduct DE analyses. limma utilizes a standard variance moderated across all genes using a Bayesian model and produces *P*-values with greater degrees of freedom (55). The voom module was used to transform the data based on observational level weights derived from the mean-variance relationship prior to statistical modeling (56) (Supplementary Figure S2). Experimental batch effects were adjusted for by including experimental batch as a covariate in the statistical model (57). Differentially expressed genes were defined as genes with a Benjamini–Hochberg multiple-testing adjusted *P*-value of <0.05.

### Gene Ontology (GO) analysis

Enriched Gene Ontology (GO) categories were identified using the GOseq package in R (58). GOseq was developed specifically to account for transcript length bias in GO analyses using RNA-seq data. Two gene sets were input separately into GOseq: all genes previously identified as upregulated in metacyclic promastigotes and all genes previously identified as downregulated in metacyclic promastigotes (see Dataset S2). A *P*-value cut-off of <0.05 was used.

### *Trans*-splicing site detection and 5′ UTR analysis

Sequences from each sample were mapped to the *L. major* genome (v. 6.0) using TopHat (v 2.0.10) (50). Only one mismatch per read was allowed and the paired reads were required to be mapped for an alignment to be reported. Reads that did not align to the genome were retained to form a pool of candidate SL-containing reads. These reads were filtered to keep only those containing at least four bases of the end of the *L. major* SL sequence (AACTAACGCTATTATTGATACAGTTTCTGTACTATATTG) or its reverse complement. This target sequence (or its reverse complement) was trimmed from the reads and TopHat was used to align the remaining portions to the *L. major* genome. Two mismatches per read were allowed and reads were assigned only to a single locus of the gene model annotations provided to TopHat (containing previously annotated genes and novel ORFs). The alignment coordinates of the trimmed reads were used to retrieve the exact locations of the putative *trans*-splicing sites. The genomic sequence neighboring each putative site was compared against the portion of the read that was removed. Reads for which the trimmed portion (4–39 nt) did not differ by at least two bases from the corresponding genomic sequence were treated as false hits and discarded. Putative sites that were located within a previously annotated coding sequence (CDS) (from TriTrypDB, version 6.0) or within a novel ORF (as defined above) or those with no such feature within 7500 nt downstream of the site were excluded. *Trans*-splicing sites that remained were assigned to the nearest downstream feature. The length of the 5′ UTR was defined as the distance between the *trans*-splicing site and the start of the CDS/ORF to which it was assigned. Splice acceptor sites were identified for each gene by extracting the dinucleotide sequence in the genome upstream of each detected *trans*-splicing site using a custom Python script. Sequence composition was plotted using WebLogo version 3.3 (59). The *trans*-splicing site detection pipeline was written in Python and made use of the Ruffus pipeline software framework (60) and Biopython library (61). Data visualization was done using ggplot2 (62).

### Polypyrimidine tract characterization

A custom Python script was used to scan a window of 250 nt upstream of each primary *trans*-splicing site to identify the corresponding polypyrimidine (polyPy) tract. A polyPy tract was defined as the longest stretch of sequence consisting of pyrimidines, allowing interruption by no more than a single purine.

### Polyadenylation site detection and 3′ UTR analysis

Identification of the polyadenylation sites was done using a process similar to the one used for *trans*-splicing site detection. The initial filtering step performed on unmapped reads identified reads containing at least 4 nt of thymine or at least 4 nt of adenine residues. This target sequence was trimmed from the reads and TopHat was used to align the remaining portions of the reads to the *L. major* genome. Two mismatches per read were allowed and reads were assigned only to a single locus in the gene model annotations provided to TopHat (containing previously annotated genes and novel ORFs). The alignment coordinates of the trimmed reads were used to retrieve the exact locations of the putative polyadenylation sites. The sequence neighboring each putative site was compared against the portion of the read that was removed. Any reads for which the trimmed portion (4+ nt) did not differ by at least two bases from the corresponding genomic sequence were treated as false hits and discarded. Putative sites that were located within a previously annotated CDS (from TriTrypDB, version 6.0) or within a novel ORF (as defined above) or those with no such feature within 7500 nt upstream of the site were excluded. Polyadenylation sites that remained were assigned to the nearest downstream feature (CDS or novel ORF). The length of the 3′ UTR was defined as the distance between the stop of the CDS/ORF and the polyadenylation site. Sequence composition was plotted using WebLogo version 3.3 (59). The polyadenylation site detection pipeline was written in Python and made use of the Ruffus pipeline software framework (60) and Biopython library (61). Data visualization was done using ggplot2 (62).

### Alternative RNA processing site analysis

Counts of *trans*-splicing sites or polyadenylation sites were combined from biological replicates for each developmental stage (procyclic promastigotes and metacyclic promastigotes). The site with the largest number of reads mapped was defined as ‘primary’ for each of the developmental stages. All other sites were considered to be ‘minor’ with the most utilized of the minor sites designated as the ‘secondary’ site. The ratio of reads mapping to the primary site to those mapping to the secondary site (P/S) for a given gene was used to determine the dominance (preference) of the primary site for that gene.

### Data access

Sequence data are available at the NCBI sequence read archive (SRA) under accession numbers SRR1460763-SRR1460775. All components of the data quality assessment statistical pipeline, named cbcbSEQ, were done in R and can be accessed on GitHub (https://github.com/kokrah/cbcbSEQ/). The code used for the *trans*-splicing and polyadenylation pipelines is freely available at https://github.com/elsayed-lab/utr_analysis. The code used to determine alternative RNA processing sites is available at https://github.com/elsayed-lab/lmajor_alternate_acceptor_site_usage.

## RESULTS

### Experimental design

Transcriptome profiling by RNA-seq was used to identify global changes in gene expression as *L. major* achieves infectivity. RNA was isolated from cultured *L. major* grown to log phase (procyclic form) or enriched for metacyclic forms using: (i) a Ficoll gradient or (ii) negative selection using PNA. These two methods for metacyclic promastigote enrichment were used to test whether different methods for the procurement of metacyclic parasites could be responsible for different findings in previous studies (35,37,39,43). PolyA enriched cDNA libraries were generated using the Illumina TruSeq protocol and 100-bp paired end sequences were generated. A total of six procyclic promastigote biological replicates and nine metacyclic promastigote biological replicates were collected (Supplementary Table S1). Each procyclic replicate was matched to one or two metacyclic replicates from the same batch/expansion of cells. Phase contrast images, promastigote sample quantification and an infectivity curve for the parasites in murine macrophages are provided in Supplementary Figure S3.

A total of ∼1.1 billion sequence reads were produced across the 15 samples, 91% of which mapped to the *L. major* reference genome (Supplementary Table S1). For each sample, the number of reads mapping to existing gene annotations was determined. The resulting count table was restricted to the 8486 protein-coding genes in the TriTrypDB *L. major* annotation v. 6.0.

### Statistical evaluation of biological replicates and batch effects

We used multiple robust statistical methods to evaluate the global characteristics of samples and to identify outlier samples that should be removed prior to DE and gene structure analysis (Supplementary Figure S1). Box plots were used to compare the distribution of per-gene read counts within each sample. All 15 samples showed a similar distribution of these counts with median steady-state expression levels of ∼7.2 log2 counts per million and very few genes (5–10 per sample) expressed at levels of <4 counts per million. This observation is consistent with a lack of gene regulation at the level of transcription and may indicate that very few protein-coding transcripts are completely degraded following polycistronic transcription. A heatmap of Pearson correlations was used to visualize the relationship between each pair of samples. While all samples showed a pairwise correlation (r) of at least 0.85, samples prepared on one experimental date (batch A) were less correlated to samples from other batches, which largely showed *r*-values of >0.95 when compared to one another. MPC was also computed to assess global correlation between samples and a standard outlier identification method was applied to establish a cut-off for the identification of outliers. Consistent with observations from the Pearson correlation heatmap analysis, this method identified the two samples from batch A as outliers. These two samples were excluded from further analyses.

The dataset used for DE analysis was further restricted to genes expressed at a level of at least 1 read per million in at least 5 of the 13 remaining samples. Of the 8486 protein-coding genes analyzed, 8475 met this threshold, consistent with observations described above that few genes were completely degraded after transcription. No statistical difference was found in protein coding gene expression between metacyclic promastigote samples prepared using the Ficoll or PNA protocols. Consequently, all metacyclic promastigote samples were pooled together for the remainder of the analyses.

The large number of biological replicates used for the analysis necessitated the evaluation of the dataset for batch effects. A batch effect represents experimental variation caused by sub-groups of measurements that are independent of the underlying biology of the system being studied. They have been shown to introduce unwanted variability into biological studies and confound the results, leading to erroneous conclusions. Previous analyses of high-throughput data, like those produced by RNA-seq, have indicated the need to assess and correct batch effects (57). In this study, we used experimental start date as a surrogate for batch when testing for DE between developmental stages of *L. major*.

**Figure 1.**
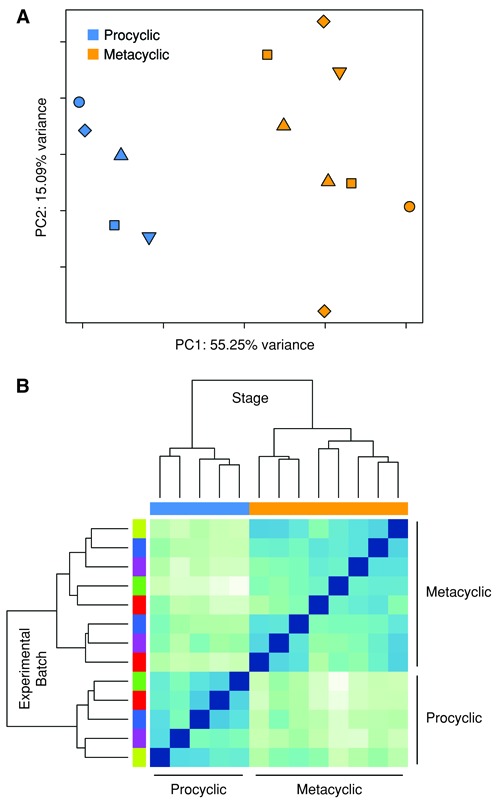
Global gene expression profiles of the procyclic and metacyclic promastigote forms of *Leishmania major*. RNA-seq was carried out on *L. major* procyclic (log phase) promastigotes and metacyclic promastigotes isolated after enrichment using Ficoll or PNA. A principal component analysis (PCA) plot (**A**) and heatmap of a hierarchical clustering analysis using the Euclidean distance metric (**B**) are shown. Both analyses were performed using all *L. major* annotated genes (8475) after filtering for low counts, quantile normalization and accounting for batch effects in the statistical model used by limma. In the PCA plot, each point represents an experimental sample with point color indicating *L. major* developmental stage (blue = procyclic promastigote, orange = metacyclic promastigote) and point shape indicating batch/experimental date. Colors along the top of the heatmap indicate the developmental stage (blue = procyclic promastigote, orange = metacyclic promastigote) and colors along the left side of the heatmap indicate the batch/experimental date.

PCA and Euclidean distance heatmap analysis were used to visualize the relationship between samples both prior to (Supplementary Figure S4) and after (Figure 1) accounting for batch effects. PCA reduces the dimensionality of a dataset while allowing variability to be represented to the greatest extent possible (63). The PCA plots showed the first two principal components, which account for the greatest percent of variability in the data, on the X and Y axes, respectively, with each of the 13 samples represented as a single point. When batch was accounted for, a clear separation between procyclic promastigote and metacyclic promastigote samples was seen along the X axis of the PCA plot (Figure 1A). Separation between the stages was not as pronounced when batch was not considered (Supplementary Figure S4A). Indeed, prior to accounting for batch effects, 25% of the variance represented by PC1 and 77% of the variance represented by PC2 were attributable to the batch of the samples. Likewise, when Euclidean distance between samples was computed and used to create a heatmap color image and dendrogram depicting the closeness between samples, a clear separation between procyclic promastigote and metacyclic promastigote samples was observed after accounting for batch effects (Figure 1B) but not before (Supplementary Figure S4B). As a result of these analyses, batch effects were controlled for in the subsequent DE analysis by including experimental batch in the statistical model used by limma.

### Identification of genes differentially expressed between developmental stages

**Figure 2.**
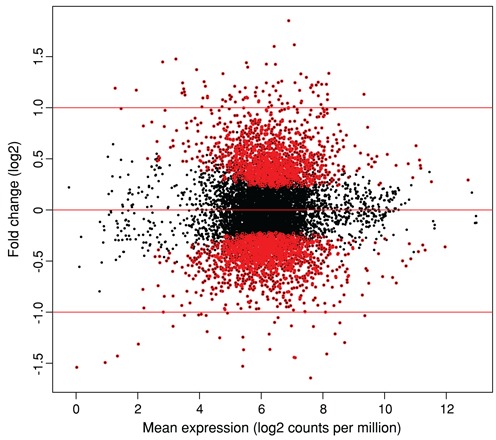
The differentiation of *Leishmania major* from the procyclic form to the infective metacyclic form reveals global changes in the parasite's gene expression program. RNA-seq was carried out on *L. major* procyclic and metacyclic promastigotes. DE analysis was done using limma after voom transformation, taking experimental batch into account as part of the limma statistical model. The MA plot shows the relationship between mean expression (log2 counts per million with an offset of 0.5) and fold change. Each point represents one gene. Points colored in red represent 3138 genes expressed at significantly different levels between procyclic and metacyclic promastigotes at an adjusted *P*-value of <0.05, with genes upregulated in the metacyclic stage relative to the procyclic stage exhibiting positive fold changes.

DE analysis identified 3138 genes that were expressed at significantly different levels between procyclic and metacyclic promastigotes at an adjusted *P*-value cutoff of <0.05 (Dataset S2). Fold change differences ranged from 3.1-fold downregulated to 3.6-fold upregulated in metacyclic promastigotes. These genes were visualized using an MA plot showing the relationship between mean expression and fold change for each gene (Figure 2). Almost 60% of the DE genes (1829 of 3138) are annotated as hypothetical proteins. The remaining gene products have been characterized to different extents, albeit not always in the context of their possible role(s) in metacyclogenesis.

We extended our DE analysis to a set of 1044 novel ORFs of at least 90 nt in length identified based on evidence of translation in *L. major* by ribosome profiling data (Dataset S1). The addition of these ORFs lead to an increase in the list of differentially expressed genes by ∼12% (a total of 368, from 3138 to 3506) with fold changes ranging from 5.7-fold downregulated to 3.6-fold upregulated in the metacyclic stage (Dataset S3). The top 25 down- and upregulated genes are shown in Table 1. Of these, 8 are novel ORFs, including the most downregulated gene.

**Table 1.** Top 25 differentially expressed genes in the *L. major* procyclic to metacyclic promastigote transition

	**Metacyclic promastigotes, downregulated**	
**ID**	**Product description**	**Fold change**
LmjF.23_3931	novel ORF, LmjF.23, 477090–477218 (−)	5.65
LmjF.31.3070	iron/zinc transporter protein-like protein (LIT1)	3.14
LmjF.35.1310	histone H4	2.90
LmjF.36.0020	histone H4	2.74
LmjF.35.2160	adenine aminohydrolase (AAH)	2.72
LmjF.31.3180	histone H4	2.68
LmjF.33.1760	hypothetical protein, unknown function	2.66
LmjF.14.0470	hypothetical protein, conserved	2.65
LmjF.21.0740	ATPase subunit 9, putative	2.57
LmjF.35.2130	hypothetical protein, unknown function	2.56
LmjF.33.3240	h1 histone-like protein	2.56
LmjF.25.2450	histone H4	2.48
LmjF.32_7004	novel ORF, LmjF.32, 1161019-1161147 (−)	2.39
LmjF.36.5845	kinetoplast-associated protein, putative	2.37
LmjF.36.3080	lipoate protein ligase, putative	2.35
LmjF.35.4760	hypothetical protein, conserved	2.34
LmjF.02.0020	histone H4	2.34
LmjF.32.2940	hypothetical protein, conserved	2.33
LmjF.23.0200	endoribonuclease L-PSP (Pb5), putative	2.22
LmjF.35_8354	novel ORF, LmjF.35, 877847-877972 (+)	2.20
LmjF.25.1470	cyclin (CYCA)	2.20
LmjF.20.0030	histone-lysine N-methyltransferase, putative (DOT1)	2.20
LmjF.19_3054	novel ORF, LmjF.19, 382655-382816 (+)	2.16
LmjF.13_1846	novel ORF, LmjF.13, 171578-171685 (+)	2.16
LmjF.36.3910	S-adenosylhomocysteine hydrolase	2.16

	**Metacyclic promastigotes, upregulated**	
**ID**	**Product description**	**Fold change**
LmjF.34.0070	ascorbate peroxidase (APX)	3.61
LmjF.19_3059	novel ORF, LmjF.19, 395719-395889 (+)	3.31
LmjF.02.0460	voltage-dependent anion-selective channel, putative	3.04
LmjF.17.0890	META domain containing protein (META1)	3.03
LmjF.23.0730	RNA-binding protein, putative	2.97
LmjF.12.0480	hypothetical protein, unknown function	2.95
LmjF.28.0980	P27 protein, putative (P27)	2.77
LmjF.23.0780	hypothetical protein, conserved	2.68
LmjF.29.1350	RNA binding protein, putative	2.68
LmjF.16.0500	hypothetical protein, unknown function	2.68
LmjF.22.0250	phosphoinositide phosphatase	2.63
LmjF.29.1360	RNA binding protein, putative	2.63
LmjF.36.2290	serine/threonine protein kinase, putative	2.61
LmjF.34.1940	amastin-like surface protein, putative	2.54
LmjF.17_2659	novel ORF, LmjF.17, 423627–423884 (+)	2.53
LmjF.04.0350	hypothetical protein, conserved	2.52
LmjF.16.1050	hypothetical protein, conserved	2.52
LmjF.34.2500	protein phosphatase 2C-like protein	2.49
LmjF.35.5000	hypothetical protein, conserved	2.46
LmjF.04.1210	casein kinase I, putative	2.45
LmjF.34.1820	amastin-like surface protein, putative	2.44
LmjF.12.0460	hypothetical protein, unknown function	2.43
LmjF.09_1121	novel ORF, LmjF.09, 124955–125314 (+)	2.41
LmjF.34.1800	amastin-like surface protein, putative	2.40
LmjF.17.0630	hypothetical protein, unknown function	2.40

A total of 3506 previously annotated genes and novel ORFs were differentially expressed (DE) between procyclic and metacyclic promastigotes at an adjusted *P*-value of <0.05 (no fold change cut-off, 127 genes with two-fold cut-off). The top 25 down- and upregulated genes/novel ORFs are shown. Gene identifiers containing an underscore character correspond to novel ORFs as listed in Dataset S1.

The list of DE genes was used as input into GO analysis to identify cellular functions and processes that are enriched during *L. major* metacyclogenesis. Genes downregulated in metacyclic promastigotes were considered separately from upregulated genes. Forty GO categories were identified as being significantly enriched (*P*-value cutoff of <0.05) for genes downregulated (33 categories) and upregulated (7 categories) in metacyclic promastigotes (Table 2 and Dataset S4).

**Table 2.** Gene ontology (GO) categories enriched during the procyclic to metacyclic transition

	**Metacyclic promastigotes, downregulated**	
**GO ID**	**GO term**	***P*-value**
GO:0015986	ATP synthesis coupled proton transport	7.36e-11
GO:0005737	cytoplasm	5.33e-09
GO:0015991	ATP hydrolysis coupled proton transport	2.00e-08
GO:0046961	proton-transporting ATPase activity, rotational mechanism	2.79e-08
GO:0004812	aminoacyl-tRNA ligase activity	6.96e-08
GO:0006418	tRNA aminoacylation for protein translation	6.96e-08
GO:0046933	proton-transporting ATP synthase activity, rotational mechanism	7.26e-08
GO:0044267	cellular protein metabolic process	1.41e-07
GO:0003746	translation elongation factor activity	1.55e-07
GO:0005634	nucleus	3.38e-07
GO:0006260	DNA replication	1.25e-06
GO:0005525	GTP binding	1.42e-06
GO:0003677	DNA binding	1.94e-06
GO:0003924	GTPase activity	2.64e-06
GO:0043234	protein complex	1.64e-05
GO:0051258	protein polymerization	1.64e-05
GO:0003743	translation initiation factor activity	1.74e-05
GO:0045261	proton-transporting ATP synthase complex, catalytic core F(1)	5.23e-05
GO:0006457	protein folding	7.78e-05
GO:0051082	unfolded protein binding	8.59e-05
GO:0005874	microtubule	1.07e-04
GO:0004298	threonine-type endopeptidase activity	1.37e-04
GO:0005839	proteasome core complex	1.37e-04
GO:0051603	proteolysis involved in cellular protein catabolic process	1.37e-04
GO:0006334	nucleosome assembly	1.47e-04
GO:0006413	translational initiation	1.93e-04
GO:0046982	protein heterodimerization activity	2.60e-04
GO:0003887	DNA-directed DNA polymerase activity	3.45e-04
GO:0006414	translational elongation	6.65e-04
GO:0005198	structural molecule activity	9.43e-04
GO:0004175	endopeptidase activity	9.86e-04
GO:0016272	prefoldin complex	1.07e-03
GO:0050660	flavin adenine dinucleotide binding	1.09e-03

	**Metacyclic promastigotes, upregulated**	
**GO ID**	**GO term**	***P*-value**
GO:0004674	protein serine threonine kinase activity	4.61e-22
GO:0006468	protein phosphorylation	1.91e-21
GO:0004672	protein kinase activity	8.67e-20
GO:0004713	protein tyrosine kinase activity	1.28e-18
GO:0005524	ATP binding	3.82e-11
GO:0006950	response to stress	2.77e-10
GO:0016791	phosphatase activity	1.63e-04

GOseq (58) was used to perform gene ontology analysis using differentially expressed genes identified as the parasite undergoes metacyclogenesis. Using a *P*-value cut off of <0.05, a total of 33 GO categories were enriched among genes that were downregulated in metacyclic promastigotes and a total of seven GO categories were enriched among genes that were upregulated in metacyclic promastigotes. The differentially expressed genes corresponding to each enriched GO category are reported in Dataset S4.

### Examination of differentially expressed gene lists and gene ontology-based enrichment analyses

Many novel genes were identified among the most downregulated during metacyclogenesis, including multiple genes with unknown function. GO enrichment analysis of these genes reflected a clear reduction in a number of cellular processes including DNA replication and nucleosome assembly, translation-related activities (initiation and elongation), protein metabolism and energy metabolism (i.e. adenosine triphosphate; ATP synthesis) while enriched GO categories for genes upregulated in metacyclic promastigotes indicated an increase in cell signaling and stress response (Table 2, Supplementary Figure S5 and Dataset S4).

A close examination of differentially expressed genes and genome ontology enrichments confirmed earlier findings and, more importantly, revealed new insights into the parasite's transformation at a critical stage of its life cycle. The top downregulated gene in metacyclic promastigotes, LIT1 (LmjF.31.3070) is an iron transporter previously reported to be upregulated by the parasite upon iron depletion (64). Its downregulation in metacyclics is consistent with the low metabolic rate and low demand for ATP in this developmental stage of the parasite. Interestingly, its paralogous copy (LmjF.31.3060) was regulated to a lesser extent (downregulated only ∼1.5-fold in metacyclics). Multiple histones (H2A, H2B, H4 and H1 histone-like protein) previously identified as downregulated during metacyclogenesis (65,66) were also identified as such in this analysis with H4 mRNA levels particularly depleted. The decrease in histone transcripts as the parasite enters the non-dividing stationary phase suggests a mode of regulation that is dependent on the cell cycle and is consistent with observations in higher eukaryotes that histone gene expression decreases in differentiated cells (67,68). Also consistent with previous findings, multiple β-tubulin family members were identified as downregulated ∼1.4-fold as the parasite becomes infective (69). The downregulation of β-tubulin as the parasite undergoes metacyclogenesis correlates with morphological changes of the parasite as it prepares to enter host cells. Additionally, the steady-state RNA level for adenine aminohydrolase (AAH), a purine metabolism protein that converts adenine to hypoxanthine and lacks homologs in humans as well as *T. cruzi* and *T. brucei* (70), was found to be reduced in *L. major* metacyclic promastigotes in our study, as were cyclin A and DOT1, which are both involved in cell cycle progression (71,72).

The top upregulated gene, ascorbate peroxidase, is protective against both endogenous and exogenous H_2_O_2_ and appears to play a role in differentiation to the metacyclic form as well as in protecting the cell against oxidative stress-induced apoptosis (73). Other genes that were upregulated in metacyclic promastigotes include casein kinase 1, a Ser/Thr protein kinase that exists in multiple isoforms and has been identified as playing a role in *Leishmania* infectivity (74), and *meta1*, which encodes a protein that localizes in the region of the flagellar pocket of stationary phase promastigotes and is thought to play a role in virulence, potentially through altering secretory processes (75,76). The *p27* gene, which encodes a mitochondrial membrane protein that is an important component of the cytochrome oxidase complex, was also more abundantly expressed in metacyclic promastigotes. This result is consistent with previous findings reporting its upregulation in both metacyclics and intracellular amastigotes and its role in promoting parasite survival and virulence in the host (77) and supports the hypothesis that metacyclic promastigotes are pre-adapted to survival within the mammalian host (78). Finally, two known differentiation markers of metacyclic promastigotes, SHERP and HASPB (79–81), were also identified in this analysis, with SHERP upregulated ∼1.9-fold and HASPB upregulated ∼2.3-fold in metacyclic promastigotes.

The results of the DE analysis were compared to the list of differentially expressed genes identified in an earlier study by Saxena *et al*. (43) that used microarrays of PCR-amplified fragments from genomic survey sequence (GSS) clones. Only GSS clones whose 5′ and 3′ sequences could be mapped to the same gene in the *L. major* Friedlin genome sequence (31 in total) were considered in our comparison and 19 of the corresponding genes showed a similar DE trend, albeit to varying degrees and levels of significance. Given the disparate platforms, the level of agreement was reasonable.

### Identification of transcript boundaries

Deep sequencing of *L. major* procyclic and metacyclic promastigote samples by RNA-seq presented an opportunity to comprehensively annotate transcript boundaries, thereby enhancing the structural annotation of *L. major* genes. We exploited the signal sequences generated by *trans*-splicing and polyadenylation events to accurately map the 5′ and 3′ UTR boundaries of transcripts by comparing reads containing these signals to the reference genome sequence. Since UTRs are expected to contain motifs that direct the post-transcriptional regulation of individual mRNAs—including degradation, storage and translation rate—determining transcript boundaries is very important for understanding gene regulation in the parasite.

Distinct transcript boundaries were determined for a large majority of previously annotated protein-coding genes and novel genes for which there was evidence of translation by ribosome profiling (Dataset S1). To do this, RNA-seq reads which did not map to the *L. major* genome due to RNA processing events were examined separately for evidence of SL sequence and a polyA tail. Of the ∼960 million reads from the 13 *L. major* samples, ∼3.9% contained evidence of *trans*-splicing and ∼0.05% contained evidence of polyadenylation (Supplementary Table S1). Once the SL and polyA sequences were removed, the remainders of the reads were mapped to the genome, allowing the identification of coordinates for at least one *trans*-splicing site for 8981 genes (94.2% of a total of 9530 genes) and at least one polyadenylation site for 8841 genes (92.8%). The coordinates of all identified *trans*-splicing and polyadenylation sites are provided in Dataset S5.

A sampling of the *trans*-splicing and polyadenylation sites identified here was compared to existing data in TriTrypDB (Peter Myler's group, Seattle Biomed) that were generated using an RNA-seq method that specifically enriched for SL-containing sequences (biological sample type unknown). Our *trans*-splicing site data were highly concordant with these previously reported data. This high degree of agreement is remarkable given the differences in sample type, culture and preparation across different labs and may potentially indicate that the usage of *trans*-splicing sites in *Leishmania* is fairly consistent across various biological conditions. The observed variability is likely attributable to the differences in coverage, RNA-seq approach and data analysis methodology. Our polyA site data did not generally match the existing data on TriTrypDB down to the specific nucleotide. This could be due to the extreme heterogeneity of these sites (previously reported for *T. brucei* (19)), differences in the biological samples studied or differences in the methods used to identify and assign sites.

### Gene structure features in *L. major*

**Figure 3.**
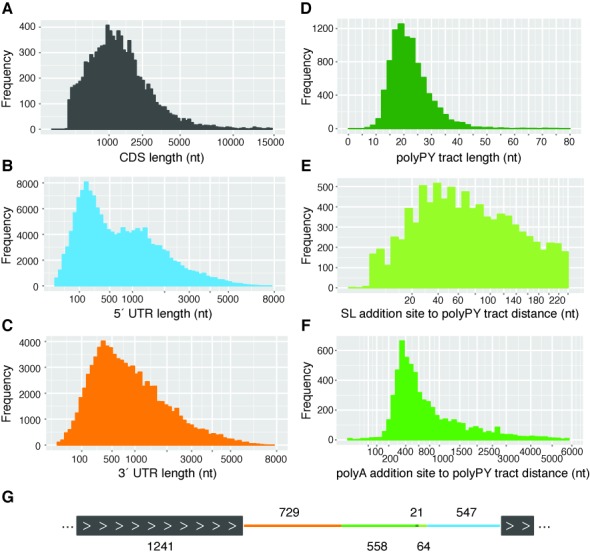
Length and position distribution of gene structure components in *Leishmania major*. Data from procyclic and metacyclic promastigote samples were combined to describe the gene structure elements of *L. major*. (**A**) Distribution of CDS lengths. Start and stop coordinates for coding sequences for previously annotated protein-coding genes (TriTrypDB v. 6.0) and novel ORFs were used to compute CDS lengths. For genes with multiple isoforms, the first isoform listed in TriTrypDB was included in the analysis. (**B**) Distribution of 5′ UTR lengths. The exact *trans*-splicing sites associated with each CDS were used to determine the coordinates and lengths of 5′ UTRs. A total of 154 046 *trans*-splicing sites were identified, corresponding to 5′ UTRs ranging from 0 to 7252 nt in length. (**C**) Distribution of 3′ UTR lengths. An analysis of polyadenylation sites associated with each CDS was performed to determine 3′ UTR coordinates and lengths. A total of 84 331 polyadenylation sites were identified, corresponding to 3′ UTRs ranging from 0 to 7133 bases in length. (**D**) Distribution of polypyrimidine (polyPy) tract lengths. A window of 250 nt upstream of the each primary *trans*-splicing site (8981 total) was scanned to identify its corresponding polyPy tract, defined as the longest stretch of pyrimidine residues interrupted by no more than one purine. (**E**) Distribution of distances between each primary *trans*-splicing site and its corresponding polypyrimidine (polyPy) tract. (**F**) Distribution of distances between each polyPy tract and the polyadenylation site of the upstream gene. A total of 6174 instances of a neighboring polyadenylation site and polyPy tract were identified. (**G**) Diagram of a ‘typical’ *L. major* genic region. The median lengths of the gene structure components of *L. major* were used to construct the structure of a ‘typical’ gene region (two genes and the intergenic region). The median values of each component are shown. Colors correspond to the features depicted in panels A–F.

We sought to determine the length distribution of the elements of each gene—5′ UTR, CDS and 3′ UTR—as well as the intergenic region, including the polypyrimidine (polyPy) tract, for previously annotated protein-coding genes and the novel ORFs. Start and stop coordinates for *L. major* genes were used to determine a median CDS length of 1241 nt with a range from 64 to 52 178 nt (Figure 3A). The boundaries of 5′ UTRs were defined using the coordinates of the SL addition sites and start codon annotations and a similar analysis was done to determine the lengths of 3′ UTRs using stop codon and polyadenylation site coordinates. The median length of all identified 5′ UTRs (not including the 39 nt SL sequence) and 3′ UTRs was 547 and 729 nt, respectively (Figure 3B and C). When only the most-utilized (primary) *trans*-splicing or polyadenylation site for each gene was considered, these values were reduced to 233 and 517 nt, respectively (see alternative RNA processing section below). The distribution of both the 5′ and 3′ UTR lengths was similar in both stages (Supplementary Figure S6) and there did not appear to be a correlation between CDS length and either UTR length or between corresponding UTR lengths.

The length distribution analysis was extended to examine the polyPy tract, which is known to be involved in the regulation of RNA processing events in trypanosomatids (82–84). In this analysis, the polyPy tract was identified as the longest stretch of pyrimidine residues located upstream of each of the (primary) *trans*-splicing sites and interrupted by no more than one purine. PolyPy tracts ranged from 7 to 123 nt in length, with a median value of 21 nt (Figure 3D) and a clear usage preference for cytosine (54%) over thymine (42%) residues. This observation, which runs counter to what has been found in related species where thymine was preferred (19,85) (Li Y, Caradonna KL, Belew AT, Corrada Bravo H, Burleigh BA, El-Sayed NM, in revision), is unsurprising given the higher GC content of *Leishmania* relative to the other trypanosomatids (5).

The median distance between each polyPy tract and its downstream SL addition site was 64 nt (Figure 3E) and the median distance between the polyPy tract and the upstream polyadenylation site (if both were detected; 6174 instances) was 558 (Figure 3F). When considering only intergenic regions that were bound by both a detectable upstream polyadenylation site and a detectable downstream SL addition site (6152 instances), a median intergenic distance of 556 nt was observed.

The median values of each gene structure element were used to determine a representative gene structure for *L. major* genes, with a median mRNA length of 2517 nt, of which the 5′ UTR, CDS and 3′ UTR account for 22, 49 and 29%, respectively (corresponding to a 5′ UTR of 547 nt, a CDS of 1241 nt and a 3′ UTR of 729 nt) (Figure 3G). The median intergenic length was 643 nt. This observed gene structure indicates significantly longer 5′ and 3′ UTRs and longer intergenic distances than what has been reported in either *T. cruzi* and *T. brucei* (19,86) (Li Y, Caradonna KL, Belew AT, Corrada Bravo H, Burleigh BA, El-Sayed NM, in revision), and is consistent with previous observations regarding the relative compactness of the species’ genomes (5).

### Detection of alternative RNA processing events within and between developmental stages

The sequencing depth of our *L. major* transcriptome profiling experiments allowed not only the identification of the SL-addition and polyadenylation sites at a single-base resolution, but also the quantification of alternative RNA processing events. Of the 8981 genes with SL-addition sites detected, 8777 (∼98%) used more than one *trans*-splicing site in at least one developmental stage. We were able to detect alternative splicing in *L. major* with a greater sensitivity than has been previously reported (45), presumably due to the deeper coverage of this dataset. Indeed, for genes with detectable *trans*-splicing events, alternative *trans*-splicing was pervasive with 88, 56 and 18% of genes using at least 5, 10 or 20 sites, respectively, in at least one developmental stage. This observation indicates that *L. major* exhibits a somewhat higher degree of alternative splicing than related species *T. cruzi* and *T. brucei* where <90% of genes were identified as alternatively spliced (19) (Li Y, Caradonna KL, Belew AT, Corrada Bravo H, Burleigh BA, El-Sayed NM, in revision). This observation persisted even after accounting for differences in sequencing depth. The distribution of the distances between the primary and minor *trans*-splicing sites revealed that almost half (∼48%) of the alternative splice sites are located within 200 bases of the primary site in either direction. Even so, a significant percentage (18%) of minor sites were observed more than 1000 bp from the primary site, with most of these (78%) occurring upstream of the primary site.

**Figure 4.**
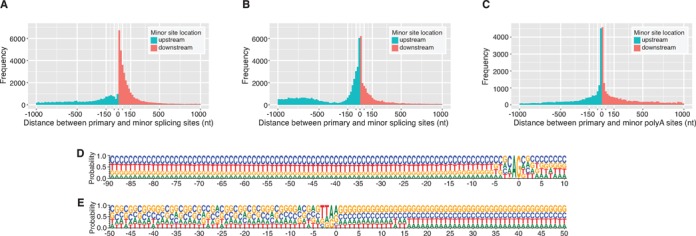
Characterization of alternative RNA processing. Alternative RNA processing events were detected in both promastigote developmental stages and pooled for this analysis. The distribution of distances between primary and minor *trans*-splicing sites is shown for minor splice sites that use (**A**) the canonical AG acceptor sequence or (**B**) an acceptor sequence other than AG. Panel (**C**) depicts the distribution of distances between primary and minor polyadenylation sites. Only minor sites within 1000 nt of the primary site are plotted. The full ranges for A, B and **C**, are −7234 to 5973, −7178 to 5876 and −6429 to 5993, respectively. About 18% (11 201 of 62 098), 29% (24 303 of 82 947) and 10% (7301 of 75 487) of values fell outside of the plotted range for A, B and C, respectively. (**D**) Sequence composition of the region spanning from 90 nt upstream to 10 nt downstream of each primary *trans*-splicing site. (**E**) Sequence composition of the region spanning from 50 nt upstream to 50 nt downstream of each primary polyadenylation site.

An examination of the *trans*-splicing sites revealed a propensity for usage of the canonical acceptor sequence (AG) at both the primary (∼97%) and minor (∼43%) splicing sites (Supplementary Table S2), consistent with previous findings in *T. cruzi* and *T. brucei* (19) (Li Y, Caradonna KL, Belew AT, Corrada Bravo H, Burleigh BA, El-Sayed NM, in revision). A sequence composition analysis of the region upstream of the SL-addition site allowed the visualization of the tail end of the polypyrimidine tract through the *trans*-splicing acceptor site (Figure 4D). As reported previously (45,87), a C nucleotide was preferred prior to the AG acceptor sequence. When considering minor sites that are located within 1 kb of the primary site, a majority (64.2%) of minor sites that use the canonical AG acceptor are located downstream of the primary site (Figure 4A). This observation supports a model (based on a study of mammalian introns) that proposes that the 3′ splice site is located by a scanning process that recognizes the first AG downstream of the branch point in a sequence-specific context (88,89). When minor sites that do not use the canonical AG acceptor sequence were considered, this phenomenon was largely absent and the percentage of minor sites that are downstream of the primary site drops to 36.3% (Figure 4B). This observation was maintained when procyclic and metacyclic promastigotes were considered separately.

Alternative polyadenylation sites were detected for 8391 (∼95%) of the 8841 genes for which polyadenylation events were observed with 61, 21 and 5% of genes using at least 5, 10 or 20 polyadenylation sites. As observed for *trans*-splicing events, this observation indicates a higher degree of alternative polyadenylation in *L. major* than what has been reported in either *T. cruzi* or *T. brucei* where 63 and 92% of genes had detectable alternative polyadenylation, respectively (Li Y, Caradonna KL, Belew AT, Corrada Bravo H, Burleigh BA, El-Sayed NM, in revision). A sequence composition analysis was done to visualize the region surrounding the polyA-addition site. Even though no consensus motif was observed upstream of the polyadenylation site, such as the AAUAAA required for polyadenylation in higher eukaryotes, we did note an (A/G)(A/G) motif preceded by 1–2 thymines abutting the polyA addition site for both primary and minor polyadenylation sites (Figure 4E). Similar to what was found for SL addition sites above, the analysis of the distribution of the distances between primary and alternative sites revealed that ∼49% of the minor polyadenylation sites were located within a 200 nt window of the primary site (Figure 4C).

**Figure 5.**
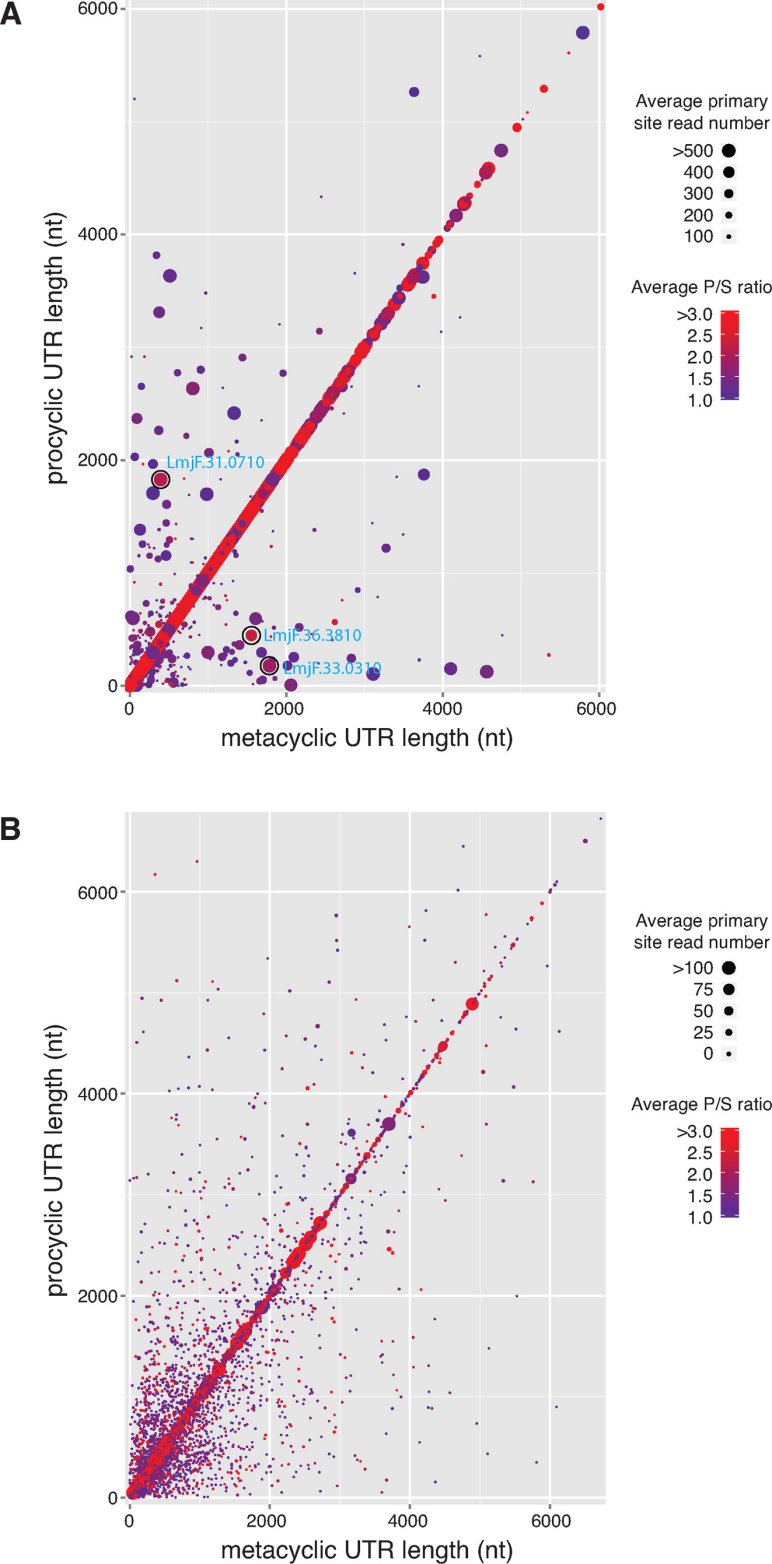
Preferential usage of primary site across developmental stages. (**A**) For each gene, the length of the 5′ UTR determined by the primary trans-splicing site for the metacyclic stage was plotted against the length of the 5′ UTR determined by the primary *trans*-splicing site for the procyclic stage. Each point represents a single gene. Points that do not fall along the diagonal represent a change in the primary site between the stages. The color of each point represents the dominance of the primary site (preference for usage of the primary site over other sites within a stage), as determined by the ratio of primary site read counts to secondary site read counts (P/S), averaged for both stages. The size of each point depicts average read count for the primary sites from both stages, thereby showing expression levels and providing a measure of confidence in the data. Three genes highlighted in Supplementary Figure S7 are circled with the gene identifier labeled in blue. (**B**) A similar plot was done for 3′ UTR length as determined by the primary polyadenylation site for each stage.

Alternative *trans*-splicing or polyadenylation are suspected to play a role in the regulation of gene expression in *L. major*, but instances of regulation through alternative RNA processing between developmental stages have not been systematically identified. We sought to identify the subset of genes that change the use of their primary *trans-*splicing or polyadenylation sites between the procyclic and metacyclic stages and to investigate possible correlations between these changes and DE. We were specifically interested in genes that showed a strong preferential usage for the primary site over other sites within a given stage (dominance), as determined using the ratio of reads that map to the primary site to those that map to the secondary site (P/S). Of the 8797 genes that had at least one *trans*-splicing site identified in both stages, 523 showed preferential usage of different primary *trans*-splicing sites between the stages. We plotted the lengths of the UTRs for each gene, as determined by the primary *trans*-splicing site in each stage (Figure 5A). Each gene was represented by a single point with the color indicating the average P/S ratio for the two stages (thereby providing a measure of a primary site's dominance) and the size indicating the average number of reads mapping to that gene's primary sites (thereby indicating expression level and an indirectly providing confidence in the data). Data points along the diagonal represent genes that did not exhibit a change in the primary *trans*-splicing site between the stages. Largely, genes that had high dominance did not exhibit a change in primary site location between the stages, but instead used the same primary site in both procyclic and metacyclic promastigotes. Genes that did change primary site tended to have only a slight preference for each stage-specific primary site. A few interesting genes did not follow this trend and showed both a change in primary site (location away from the diagonal), high dominance (red) and high confidence/expression (large). Examples of the alternative usage of *trans*-splicing sites for a subset of these interesting genes—LmjF.31.0710, LmjF.33.0310 and LmjF.36.3810—are depicted in Supplementary Figure S7.

We compared the expression profiles for genes that displayed a change in *trans*-splicing site preference versus those that did not. The proportion of differentially expressed genes in both sets was nearly identical (39.4% for genes that changed primary site and 37.0% for genes that did not), indicating that there was no association between changes in primary site used by individual genes and their expression levels (*χ*^2^ = 0.28).

A similar analysis was done to assess alternative polyadenylation between procyclic and metacyclic promastigotes (Figure 5B). Strikingly, this analysis revealed that most of the genes that showed high dominance (red) and high confidence (large) of their primary polyA sites did not exhibit a change in primary site location between the stages. For the large number of genes that showed a change in the primary polyA site between developmental stages (4377 of 8337), very few reads were mapped to the primary sites, resulting in low confidence in these data points. The low numbers of mapped polyA-containing reads was likely due to the extensive heterogeneity of polyadenylation sites or the relative low coverage of polyA-containing reads mapping at unique sites. Of the 4377 genes that changed primary polyadenylation sites between stages, the portion that was differentially expressed (37.2%) was remarkably similar to the portion that was not (38.7%; *χ*^2^ = 0.15). This lack of systematic association with DE also supports that conclusion that differences in expression levels in the samples examined by this study cannot be attributed to stage-regulated alternative RNA processing.

## DISCUSSION

The work reported here represents a comprehensive characterization of the global transcriptional changes that define the transition of the human pathogen *L. major* from its non-infective to human-infective forms. Through the exploitation of massively parallel sequencing to detect the most subtle changes in steady-state levels of mRNA, the use multiple biological replicates to derive robust statistical analyses, the careful consideration of batch effects that often confound and mask true biological effects, and the exploitation of signal sequences added during RNA processing of polycistronic pre-mRNA, we observed changes in expression profiles and identified RNA processing sites with unprecedented depth and reliability.

The genome of *L. major*, which defined the boundaries of CDS for the large majority of *L. major* genes, was completed in 2005 (6). While it has been an invaluable resource for researchers in the field, the lack of defined UTR boundaries has hampered the ability to look for sequence motifs contained in the UTRs that may be involved in the regulation of individual *L. major* genes. The RNA-seq datasets generated in this work enabled us to precisely delineate the 5′ and 3′ UTR boundaries of *L. major* transcripts, providing a substantial additional resource for the *Leishmania* research community. In addition, we were able to evaluate how transcript structure compares to related species, to examine *trans*-splicing and polyadenylation events within and between *L. major* developmental stages, and to assess the possible relationship between alternative RNA processing and gene regulation in the context of the same biological samples.

This analysis resulted in the generation of reliable and substantially deep lists of differentially expressed genes that include RNAs of low abundance, which may have fallen outside of the limits of detection in past studies. Indeed, the individual fold changes observed were relatively modest (3.1-fold downregulated to 3.6-fold upregulated in metacyclics) and may have been missed except for the sensitivity afforded by the RNA-seq technology and the statistical power provided by the use of five biological replicates in the DE analyses. The extension of the DE analysis to novel ORFs identified from ribosome profiling data points to some genes that were not included in the initial annotation of *L. major* which may have functionally important roles in the differentiation of the parasite. These genes should be added to the list of candidates to be included in future analyses.

GO analysis using the lists of differentially expressed genes revealed how they are collectively involved in a number of cellular processes as the parasite transforms into its infective form. Specifically, as the parasites become infective, processes including translation, replication and metabolism decrease while cell signaling and stress responses increase. This observation supports previous work on *Leishmania* virulence (73,75–77) and specific processes involved in metacyclogenesis, such as iron metabolism (64), cell cycle regulation (65,66,70–72) and cell signaling (74), while at the same time implicating large numbers of previously unannotated genes in these processes, thereby providing evidence as to their possible functions.

Since *Leishmania* and related trypanosomatids employ polycistronic transcription across their entire genomes, post-transcriptional RNA processing is thought to be a likely mechanism for regulating the mRNA expression levels of individual genes. While we were able to detect widespread alternative *trans*-splicing and polyadenylation for the large majority of genes, the observed heterogeneity of RNA processing sites was not systematically associated with the DE of the genes that showed the alternative RNA processing. Thus, in this analysis, RNA processing of pre-mRNA did not appear to be a driving force for determining the expression levels of individual genes.

*Trans*-splicing of a specific SL sequence is not itself unique to trypanosomatids, but has evolved in parallel in a range of diverse organisms including Euglenozoa and dinoflagellates, *Caenorhabditis elegans* and related nematodes, Platyhelminthes and primitive chordates (90). Trypanosomatids, however, are distinct because they rely on *trans*-splicing to express all genes transcribed by RNA polymerase II and because they do so without the use of any sequence-specific transcription factors. Indeed, it was this exclusive use of *trans*-splicing that enabled the precise and quantitative approach used here to define the UTR boundaries for almost all protein-coding genes. This model of transcriptional regulation differs from organisms like *C. elegans* in which ∼70% of the genes, sometimes contained in operons of functionally-related genes, are *trans*-spliced (91). While the precise role of *trans*-splicing is also unknown in *C. elegans* and other species, some evidence exists that it may play a role in responding to changes in nutrient levels (92) and that *trans*-spliced genes are enriched for Ca^2+^ homeostasis, cytoskeleton and plasma/endomembrane system function (93). Insights into RNA processing in trypanosomatids may also shed light on gene regulation in others species that rely on similar mechanisms of post-transcriptional control, even if only for a subset of genes.

In summary, transcriptome profiling of two major developmental stages of *L. major* provided a robust set of markers for the *L. major* procyclic and metacyclic developmental stages, revealed genes and processes involved in the transition between stages as the parasite becomes capable of infecting mammalian host cells, provided evidence for the function of hundred of genes of unknown function, defined precise UTR boundaries and detailed how post-transcriptional RNA processing differs between the stages. Additionally, the transcriptome profiles reported here set the stage for the construction of co-expression networks, which are useful for identifying driver mechanisms underlying co-regulation and for tentatively annotating unknown genes through guilt-by-association inferences. Our ongoing work is focused on exploring these inferences and extending our current dataset with a simultaneous interrogation of the expression profiles of the intracellular stages of *L. major* as well as its host (macrophage) cells in both murine and human systems. We have also initiated comparative transcriptome analyses of different *Leishmania* species that cause different disease outcomes. Finally, the precise definition of UTR boundaries opens up opportunities for regulatory motif analyses and comparative analyses of UTR usage across *Leishmania* species.

## Supplementary Material

SUPPLEMENTARY DATA

## References

[1] Alvar J., Vélez I.D., Bern C., Herrero M., Desjeux P., Cano J., Jannin J., den Boer M., WHO Leishmaniasis Control Team (2012). Leishmaniasis worldwide and global estimates of its incidence. PLoS One.

[2] Ambit A., Woods K.L., Cull B., Coombs G.H., Mottram J.C. (2011). Morphological events during the cell cycle of *Leishmania major*. Eukaryot. Cell.

[3] Beverley S.M., Turco S.J. (1998). Lipophosphoglycan (LPG) and the identification of virulence genes in the protozoan parasite *Leishmania*. Trends Microbiol..

[4] Wheeler R.J., Gluenz E., Gull K. (2011). The cell cycle of *Leishmania*: morphogenetic events and their implications for parasite biology. Mol. Microbiol..

[5] El-Sayed N.M., Myler P.J., Blandin G., Berriman M., Crabtree J., Aggarwal G., Caler E., Renauld H., Worthey E.A., Hertz-Fowler C. (2005). Comparative genomics of trypanosomatid parasitic protozoa. Science.

[6] Ivens A.C., Peacock C.S., Worthey E.A., Murphy L., Aggarwal G., Berriman M., Sisk E., Rajandream M.-A., Adlem E., Aert R. (2005). The genome of the kinetoplastid parasite, *Leishmania major*. Science.

[7] Martinez-Calvillo S., Yan S., Nguyen D., Fox M., Stuart K., Myler P.J. (2003). Transcription of *leishmania major* friedlin chromosome 1 initiates in both directions within a single region. Mol. Cell.

[8] Sutton R.E., Boothroyd J.C. (1986). Evidence for *Trans* splicing in trypanosomes. Cell.

[9] Lidie K.B., van Dolah F.M. (2007). Spliced leader RNA-mediated *trans*-splicing in a dinoflagellate, *Karenia brevis*. J. Eukaryot. Microbiol..

[10] Zhang H., Hou Y., Miranda L., Campbell D.A., Sturm N.R., Gaasterland T., Lin S. (2007). Spliced leader RNA *trans*-splicing in dinoflagellates. Proc. Natl. Acad. Sci. U.S.A..

[11] Krause M., Hirsh D. (1987). A *trans*-spliced leader sequence on actin mRNA in *C. elegans*. Cell.

[12] Vandenberghe A.E., Meedel T.H., Hastings K.E. (2001). mRNA 5′-leader *trans*-splicing in the chordates. Genes Dev..

[13] Ganot P., Kallesøe T., Reinhardt R., Chourrout D., Thompson E.M. (2004). Spliced-leader RNA *trans* splicing in a chordate, *Oikopleura dioica*, with a compact genome. Mol. Cell. Biol..

[14] McDonagh P.D., Myler P.J., Stuart K. (2000). The unusual gene organization of *Leishmania major* chromosome 1 may reflect novel transcription processes. Nucleic Acids Res..

[15] Myler P.J., Audleman L., deVos T., Hixson G., Kiser P., Lemley C., Magness C., Rickel E., Sisk E., Sunkin S. (1999). *Leishmania major* Friedlin chromosome 1 has an unusual distribution of protein-coding genes. Proc. Natl. Acad. Sci. U.S.A..

[16] LeBowitz J.H., Smith H.Q., Rusche L., Beverley S.M. (1993). Coupling of poly (A) site selection and *trans*-splicing in *Leishmania*. Genes Dev..

[17] Matthews K.R., Tschudi C., Ullu E. (1994). A common pyrimidine-rich motif governs *trans*-splicing and polyadenylation of tubulin polycistronic pre-mRNA in trypanosomes. Genes Dev..

[18] Ullu E., Matthews K.R., Tschudi C. (1993). Temporal order of RNA-processing reactions in trypanosomes: rapid *trans* splicing precedes polyadenylation of newly synthesized tubulin transcripts. Mol. Cell. Biol..

[19] Kolev N.G., Franklin J.B., Carmi S., Shi H., Michaeli S., Tschudi C. (2010). The transcriptome of the human pathogen *Trypanosoma brucei* at single-nucleotide resolution. PLoS Pathog..

[20] Brittingham A., Miller M.A., Donelson J.E., Wilson M.E. (2001). Regulation of GP63 mRNA stability in promastigotes of virulent and attenuated *Leishmania chagasi*. Mol. Biochem. Parasitol..

[21] Charest H., Zhang W.W., Matlashewski G. (1996). The developmental expression of *Leishmania donovani* A2 amastigote-specific genes is post-transcriptionally mediated and involves elements located in the 3′-untranslated region. J. Biol. Chem..

[22] Fadda A., Ryten M., Droll D., Rojas F., Färber V., Haanstra J.R., Merce C., Bakker B.M., Matthews K., Clayton C. (2014). Transcriptome-wide analysis of trypanosome mRNA decay reveals complex degradation kinetics and suggests a role for co-transcriptional degradation in determining mRNA levels. Mol. Microbiol..

[23] Folgueira C., Quijada L., Soto M., Abanades D.R., Alonso C., Requena J.M. (2005). The translational efficiencies of the two *Leishmania infantum* HSP70 mRNAs, differing in their 3′-untranslated regions, are affected by shifts in the temperature of growth through different mechanisms. J. Biol. Chem..

[24] Manful T., Fadda A., Clayton C. (2011). The role of the 5′-3′ exoribonuclease XRNA in transcriptome-wide mRNA degradation. RNA.

[25] Michaeli S. (2011). *Trans*-splicing in trypanosomes: machinery and its impact on the parasite transcriptome. Future Microbiol..

[26] Peacock C.S., Seeger K., Harris D., Murphy L., Ruiz J.C., Quail M.A., Peters N., Adlem E., Tivey A., Aslett M. (2007). Comparative genomic analysis of three *Leishmania* species that cause diverse human disease. Nat. Genet..

[27] Rogers M.B., Hilley J.D., Dickens N.J., Wilkes J., Bates P.A., Depledge D.P., Harris D., Her Y., Herzyk P., Imamura H. (2011). Chromosome and gene copy number variation allow major structural change between species and strains of *Leishmania*. Genome Res..

[28] Zilka A., Garlapati S., Dahan E., Yaolsky V., Shapira M. (2001). Developmental regulation of heat shock protein 83 in *Leishmania*. 3′ processing and mRNA stability control transcript abundance, and translation id directed by a determinant in the 3′-untranslated region. J. Biol. Chem..

[29] Coughlin B.C., Teixeira S.M., Kirchhoff L.V., Donelson J.E. (2000). Amastin mRNA abundance in *Trypanosoma cruzi* is controlled by a 3′-untranslated region position-dependent *cis*-element and an untranslated region-binding protein. J. Biol. Chem..

[30] Quijada L., Soto M., Alonso C., Requena J.M. (2000). Identification of a putative regulatory element in the 3′-untranslated region that controls expression of HSP70 in *Leishmania infantum*. Mol. Biochem. Parasitol..

[31] Mair G., Shi H., Li H., Djikeng A., Aviles H.O., Bishop J.R., Falcone F.H., Gavrilescu C., Montgomery J.L., Santori M.I. (2000). A new twist in trypanosome RNA metabolism: *cis*-splicing of pre-mRNA. RNA.

[32] Clayton C., Shapira M. (2007). Post-transcriptional regulation of gene expression in trypanosomes and leishmanias. Mol. Biochem. Parasitol..

[33] Berriman M., Ghedin E., Hertz-Fowler C., Blandin G., Renauld H., Bartholomeu D.C., Lennard N.J., Caler E., Hamlin N.E., Haas B. (2005). The genome of the African trypanosome *Trypanosoma brucei*. Science.

[34] El-Sayed N.M., Myler P.J., Bartholomeu D.C., Nilsson D., Aggarwal G., Tran A.-N., Ghedin E., Worthey E.A., Delcher A.L., Blandin G. (2005). The genome sequence of *Trypanosoma cruzi*, etiologic agent of Chagas disease. Science.

[35] Akopyants N.S., Matlib R.S., Bukanova E.N., Smeds M.R., Brownstein B.H., Stormo G.D., Beverley S.M. (2004). Expression profiling using random genomic DNA microarrays identifies differentially expressed genes associated with three major developmental stages of the protozoan parasite *Leishmania major*. Mol. Biochem. Parasitol..

[36] Cohen-Freue G., Holzer T.R., Forney J.D., McMaster W.R. (2007). Global gene expression in *Leishmania*. Int. J. Parasitol..

[37] Depledge D.P., Evans K.J., Ivens A.C., Aziz N., Maroof A., Kaye P.M., Smith D.F. (2009). Comparative expression profiling of *Leishmania*: modulation in gene expression between species and in different host genetic backgrounds. PLoS Negl. Trop. Dis..

[38] Gregory D.J., Sladek R., Olivier M., Matlashewski G. (2008). Comparison of the effects of *Leishmania major* or *Leishmania donovani* infection on macrophage gene expression. Infect. Immun..

[39] Guerfali F.Z., Laouini D., Guizani-Tabbane L., Ottones F., Ben-Aissa K., Benkahla A., Manchon L., Piquemal D., Smandi S., Mghirbi O. (2008). Simultaneous gene expression profiling in human macrophages infected with *Leishmania major* parasites using SAGE. BMC Genomics.

[40] Holzer T.R., McMaster W.R., Forney J.D. (2006). Expression profiling by whole-genome interspecies microarray hybridization reveals differential gene expression in procyclic promastigotes, lesion-derived amastigotes, and axenic amastigotes in *Leishmania mexicana*. Mol. Biochem. Parasitol..

[41] Rochette A., Raymond F., Ubeda J.-M., Smith M., Messier N., Boisvert S., Rigault P., Corbeil J., Ouellette M., Papadopoulou B. (2008). Genome-wide gene expression profiling analysis of *Leishmania major* and *Leishmania infantum* developmental stages reveals substantial differences between the two species. BMC Genomics.

[42] Rochette A., Raymond F., Corbeil J., Ouellette M., Papadopoulou B. (2009). Whole-genome comparative RNA expression profiling of axenic and intracellular amastigote forms of *Leishmania infantum*. Mol. Biochem. Parasitol..

[43] Saxena A., Worthey E.A., Yan S., Leland A., Stuart K.D., Myler P.J. (2003). Evaluation of differential gene expression in *Leishmania major* Friedlin procyclics and metacyclics using DNA microarray analysis. Mol. Biochem. Parasitol..

[44] Saxena A., Lahav T., Holland N., Aggarwal G., Anupama A., Huang Y., Volpin H., Myler P.J., Zilberstein D. (2007). Analysis of the *Leishmania donovani* transcriptome reveals an ordered progression of transient and permanent changes in gene expression during differentiation. Mol. Biochem. Parasitol..

[45] Rastrojo A., Carrasco-Ramiro F., Martín D., Crespillo A., Reguera R.M., Aguado B., Requena J.M. (2013). The transcriptome of *Leishmania major* in the axenic promastigote stage: transcript annotation and relative expression levels by RNA-seq. BMC Genomics.

[46] Sacks D.L., Perkins P.V. (1984). Identification of an infective stage of *Leishmania* promastigotes. Science.

[47] Späth G.F., Beverley S.M. (2001). A lipophosphoglycan-independent method for isolation of infective *Leishmania* metacyclic promastigotes by density gradient centrifugation. Exp. Parasitol..

[48] da Silva R., Sacks D.L. (1987). Metacyclogenesis is a major determinant of *Leishmania* promastigote virulence and attenuation. Infect. Immun..

[49] Bolger A.M., Lohse M., Usadel B. (2014). Trimmomatic: a flexible trimmer for Illumina sequence data. Bioinformatics.

[50] Trapnell C., Pachter L., Salzberg S.L. (2009). TopHat: discovering splice junctions with RNA-Seq. Bioinformatics.

[51] Anders S., Pyl P.T., Huber W. (2014). HTSeq-a Python framework to work with high-throughput sequencing data. Bioinformatics.

[52] Hoaglin D.C., Mosteller F., Tukey J.W. (1983). Understanding Robust and Exploratory Data Analysis.

[53] Anders S., McCarthy D.J., Chen Y., Okoniewski M., Smyth G.K., Huber W., Robinson M.D. (2013). Count-based differential expression analysis of RNA sequencing data using R and Bioconductor. Nat. Protoc..

[54] Bolstad B.M., Irizarry R.A., Astrand M., Speed T.P. (2003). A comparison of normalization methods for high density oligonucleotide array data based on variance and bias. Bioinformatics.

[55] Smyth G.K. (2004). Linear models and empirical bayes methods for assessing differential expression in microarray experiments. Stat. Appl. Genet. Mol. Biol..

[56] Law C.W., Chen Y., Shi W., Smyth G.K. (2014). voom: Precision weights unlock linear model analysis tools for RNA-seq read counts. Genome Biol..

[57] Leek J.T., Scharpf R.B., Bravo H.C., Simcha D., Langmead B., Johnson W.E., Geman D., Baggerly K., Irizarry R.A. (2010). Tackling the widespread and critical impact of batch effects in high-throughput data. Nat. Rev. Genet..

[58] Young M.D., Wakefield M.J., Smyth G.K., Oshlack A. (2010). Gene ontology analysis for RNA-seq: accounting for selection bias. Genome Biol..

[59] Crooks G.E., Hon G., Chandonia J.-M., Brenner S.E. (2004). WebLogo: a sequence logo generator. Genome Res..

[60] Goodstadt L. (2010). Ruffus: a lightweight Python library for computational pipelines. Bioinformatics.

[61] Cock P.J.A., Antao T., Chang J.T., Chapman B.A., Cox C.J., Dalke A., Friedberg I., Hamelryck T., Kauff F., Wilczynski B. (2009). Biopython: freely available Python tools for computational molecular biology and bioinformatics. Bioinformatics.

[62] Wickham H. (2009). Ggplot2 : elegant graphics for data analysis.

[63] Jolliffe I.T. (2002). Principal Component Analysis.

[64] Huynh C., Sacks D.L., Andrews N.W. (2006). A *Leishmania amazonensis* ZIP family iron transporter is essential for parasite replication within macrophage phagolysosomes. J. Exp. Med..

[65] Genske J.E., Cairns B.R., Stack S.P., Landfear S.M. (1991). Structure and regulation of histone H2B mRNAs from *Leishmania enriettii*. Mol. Cell. Biol..

[66] Soto M., Iborra S., Quijada L., Folgueira C., Alonso C., Requena J.M. (2004). Cell-cycle-dependent translation of histone mRNAs is the key control point for regulation of histone biosynthesis in *Leishmania infantum*. Biochem. J..

[67] Gerbaulet S.P., van Wijnen A.J., Aronin N., Tassinari M.S., Lian J.B., Stein J.L., Stein G.S. (1992). Downregulation of histone H4 gene transcription during postnatal development in transgenic mice and at the onset of differentiation in transgenically derived calvarial osteoblast cultures. J. Cell. Biochem..

[68] Stein J.L., van Wijnen A.J., Lian J.B., Stein G.S. (1996). Control of cell cycle regulated histone genes during proliferation and differentiation. Int. J. Obes. Relat. Metab. Disord..

[69] Coulson R.M., Connor V., Chen J.C., Ajioka J.W. (1996). Differential expression of *Leishmania major* beta-tubulin genes during the acquisition of promastigote infectivity. Mol. Biochem. Parasitol..

[70] Boitz J.M., Ullman B. (2013). Adenine and adenosine salvage in *Leishmania donovani*. Mol. Biochem. Parasitol..

[71] Hochegger H., Takeda S., Hunt T. (2008). Cyclin-dependent kinases and cell-cycle transitions: does one fit all. Nat. Rev. Mol. Cell. Biol..

[72] Kim W., Choi M., Kim J.-E. (2014). The histone methyltransferase Dot1/DOT1L as a critical regulator of the cell cycle. Cell Cycle.

[73] Pal S., Dolai S., Yadav R.K., Adak S. (2010). Ascorbate peroxidase from *Leishmania major* controls the virulence of infective stage of promastigotes by regulating oxidative stress. PLoS One.

[74] Allocco J.J., Donald R., Zhong T., Lee A., Tang Y.S., Hendrickson R.C., Liberator P., Nare B. (2006). Inhibitors of casein kinase 1 block the growth of *Leishmania major* promastigotes in vitro. Int. J. Parasitol..

[75] Nourbakhsh F., Uliana S.R., Smith D.F. (1996). Characterisation and expression of a stage-regulated gene of *Leishmania major*. Mol. Biochem. Parasitol..

[76] Puri V., Goyal A., Sankaranarayanan R., Enright A.J., Vaidya T. (2011). Evolutionary and functional insights into *Leishmania* META1: evidence for lateral gene transfer and a role for META1 in secretion. BMC Evol. Biol..

[77] Dey R., Meneses C., Salotra P., Kamhawi S., Nakhasi H.L., Duncan R. (2010). Characterization of a *Leishmania* stage-specific mitochondrial membrane protein that enhances the activity of cytochrome c oxidase and its role in virulence. Mol. Microbiol..

[78] Sacks D.L. (1989). Metacyclogenesis in *Leishmania* promastigotes. Exp. Parasitol..

[79] Flinn H.M., Rangarajan D., Smith D.F. (1994). Expression of a hydrophilic surface protein in infective stages of *Leishmania major*. Mol. Biochem. Parasitol..

[80] Knuepfer E., Stierhof Y.D., McKean P.G., Smith D.F. (2001). Characterization of a differentially expressed protein that shows an unusual localization to intracellular membranes in *Leishmania major*. Biochem. J..

[81] Sádlová J., Price H.P., Smith B.A., Votýpka J., Volf P., Smith D.F. (2010). The stage-regulated HASPB and SHERP proteins are essential for differentiation of the protozoan parasite *Leishmania major* in its sand fly vector, *Phlebotomus papatasi*. Cell. Microbiol..

[82] Günzl A. (2010). The pre-mRNA splicing machinery of trypanosomes: complex or simplified. Eukaryot. Cell.

[83] Huang J., Van der Ploeg L.H. (1991). Requirement of a polypyrimidine tract for *trans*-splicing in trypanosomes: discriminating the PARP promoter from the immediately adjacent 3′ splice acceptor site. EMBO J..

[84] Siegel T.N., Tan K.S.W., Cross G.A.M. (2005). Systematic study of sequence motifs for RNA *trans* splicing in *Trypanosoma brucei*. Mol. Cell. Biol..

[85] Greif G., Ponce de Leon M., Lamolle G., Rodriguez M., Piñeyro D., Tavares-Marques L.M., Reyna-Bello A., Robello C., Alvarez-Valin F. (2013). Transcriptome analysis of the bloodstream stage from the parasite *Trypanosoma vivax*. BMC Genomics.

[86] Siegel T.N., Hekstra D.R., Wang X., Dewell S., Cross G.A.M. (2010). Genome-wide analysis of mRNA abundance in two life-cycle stages of *Trypanosoma brucei* and identification of splicing and polyadenylation sites. Nucleic Acids Res..

[87] Requena J.M., Quijada L., Soto M., Alonso C. (2003). Conserved nucleotides surrounding the *trans*-splicing acceptor site and the translation initiation codon in *Leishmania* genes. Exp. Parasitol..

[88] Smith C.W., Porro E.B., Patton J.G., Nadal-Ginard B. (1989). Scanning from an independently specified branch point defines the 3′ splice site of mammalian introns. Nature.

[89] Smith C.W., Chu T.T., Nadal-Ginard B. (1993). Scanning and competition between AGs are involved in 3′ splice site selection in mammalian introns. Mol. Cell. Biol..

[90] Derelle R., Momose T., Manuel M., Da Silva C., Wincker P., Houliston E. (2010). Convergent origins and rapid evolution of spliced leader *trans*-splicing in metazoa: insights from the ctenophora and hydrozoa. RNA.

[91] Allen M.A., Hillier L.W., Waterston R.H., Blumenthal T. (2011). A global analysis of *C. eleganstrans*-splicing. Genome Res..

[92] Danks G.B., Raasholm M., Campsteijn C., Long A.M., Manak J.R., Lenhard B., Thompson E.M. (2014). *Trans*-splicing and operons in metazoans: translational control in maternally regulated development and recovery from growth arrest. Mol. Biol. Evol..

[93] Matsumoto J., Dewar K., Wasserscheid J., Wiley G.B., Macmil S.L., Roe B.A., Zeller R.W., Satou Y., Hastings K.E.M. (2010). High-throughput sequence analysis of *Ciona intestinalis* SL *trans*-spliced mRNAs: alternative expression modes and gene function correlates. Genome Res..

