# Supplementary material for: Transcriptomic profiling of gene expression and RNA processing during Leishmania major differentiation
Source: Nucleic Acids Res. 2015 Jul 6;43(14):6799–813. doi: 10.1093/nar/gkv656 (PMC4538839; doi:10.1093/nar/gkv656)
Supplement: SUPPLEMENTARY DATA [file supp_43_14_6799__index.html]

Transcriptomic profiling of gene expression and RNA processing during Leishmania major differentiation — Transcriptomic profiling of gene expression and RNA processing during Leishmania major differentiation — SUPPLEMENTARY DATA 

# Transcriptomic profiling of gene expression and RNA processing during *Leishmania major* differentiation

## SUPPLEMENTARY DATA

- SUPPLEMENTARY DATA
- SUPPLEMENTARY DATA
- SUPPLEMENTARY DATA
- SUPPLEMENTARY DATA
- SUPPLEMENTARY DATA
- SUPPLEMENTARY DATA
