# Supplementary material for: Transcriptomic profiling of gene expression and RNA processing during Leishmania major differentiation
Source: Nucleic Acids Res. 2015 Jul 6;43(14):6799–813. doi: 10.1093/nar/gkv656 (PMC4538839; doi:10.1093/nar/gkv656)
Supplement: SUPPLEMENTARY DATA [file supp_gkv656_nar-01429-a-2015-File009.pdf]

Supplementary Data

Transcriptomic profiling of gene expression and RNA processing during *Leishmania major* differentiation

Laura A. L. Dillon, Kwame Okrah, V. Keith Hughitt, Rahul Suresh, Yuan Li, Maria Cecilia Fernandes, A. Trey Belew, Hector Corrada Bravo, David M. Mosser, Najib M. El-Sayed

This supplement contains:

Figures S1 to S7

Tables S1 and S2

Datasets S1 to S5

Supplementary Figure Legends

Supplementary References

TABLE OF CONTENTS

Figure S1 Sample diagnostics to globally assess data similarities and identify outliers.

Figure S2 Mean-variance curve modeling and fitting of a local regression trend line by `voom`.

Figure S3 Sample characterization.

Figure S4 Principal Components Analysis (PCA) and hierarchical clustering analysis before accounting for batch effects.

Figure S5 Gene ontology trees.

Figure S6 UTR length distribution by developmental stage.

Figure S7 Visualization of changes in primary *trans*-splicing sites across developmental stages.

Table S1 Experimental design.

Table S2 Dinucleotide acceptor site usage frequency.

Dataset S1 Coordinates of novel ORFs.

Dataset S2 Results from differential expression analysis of *L. major* metacyclogenesis using `limma` (protein-coding genes from TriTrypDB v 6.0).

Dataset S3 Results from differential expression analysis of *L. major* metacyclogenesis using `limma` (protein-coding genes from TriTrypDB v 6.0 and 1,044 novel ORFs).

Dataset S4 Enriched GO categories and corresponding differentially expressed genes.

Dataset S5 UTR coordinates.

Figure S1

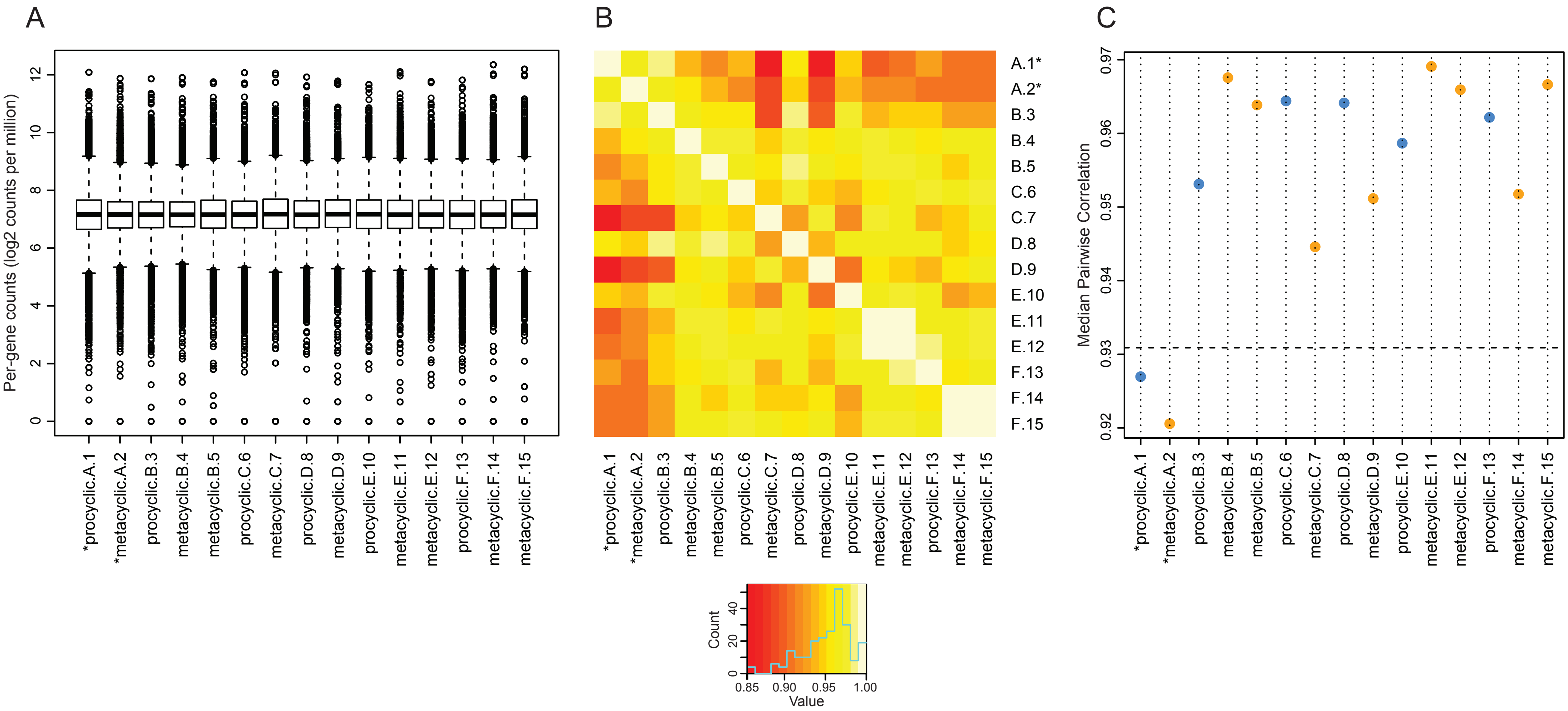
A
B
C
Per-gene counts (log2 counts per million)
Median Pairwise Correlation
Count
Value
*procyclic.A.1
*metacyclic.A.2
procyclic.B.3
metacyclic.B.4
metacyclic.B.5
procyclic.C.6
metacyclic.C.7
procyclic.D.8
metacyclic.D.9
procyclic.E.10
metacyclic.E.11
metacyclic.E.12
procyclic.F.13
metacyclic.F.14
metacyclic.F.15
A.1*
A.2*
B.3
B.4
B.5
C.6
C.7
D.8
D.9
E.10
E.11
E.12
F.13
F.14
F.15

Figure S2

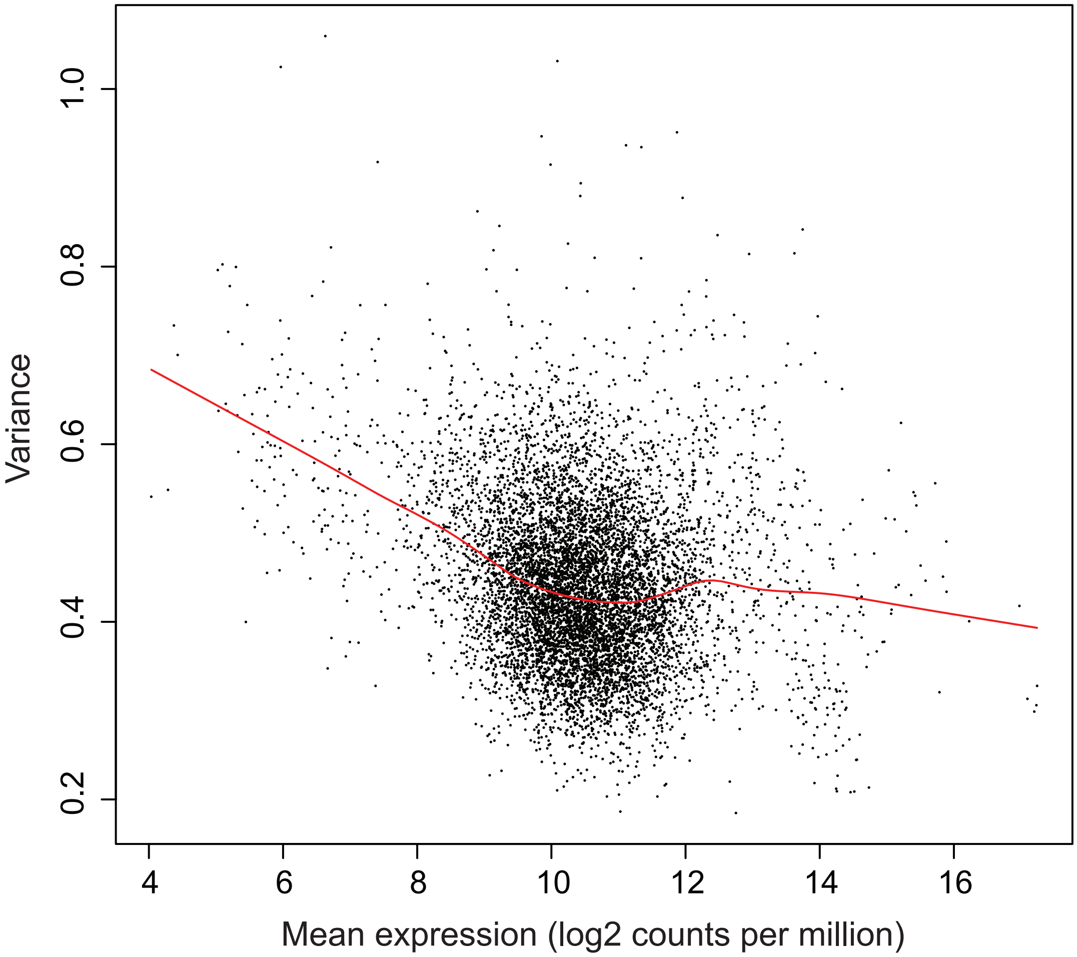

Variance
1.0
0.8
0.6
0.4
0.2
4
6
8
10
12
14
16
Mean expression (log2 counts per million)

Figure S3

A

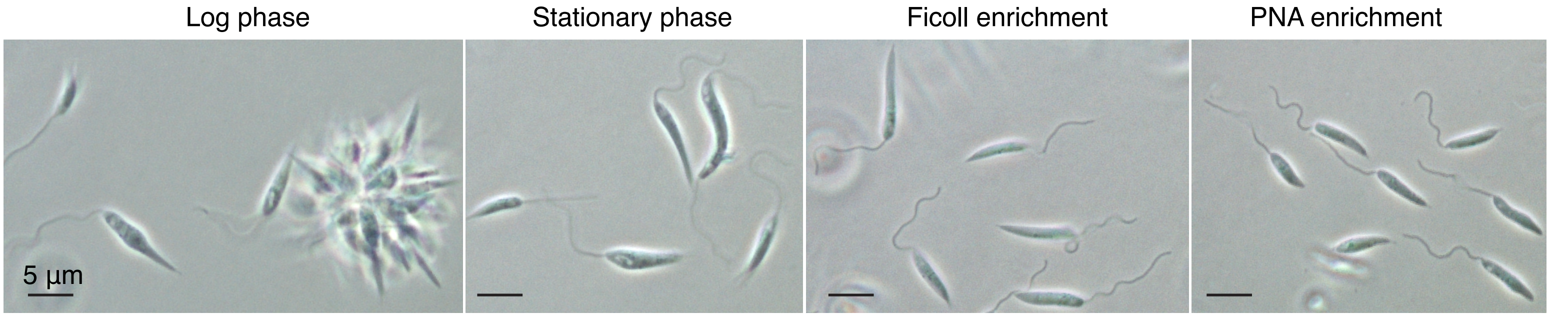

B

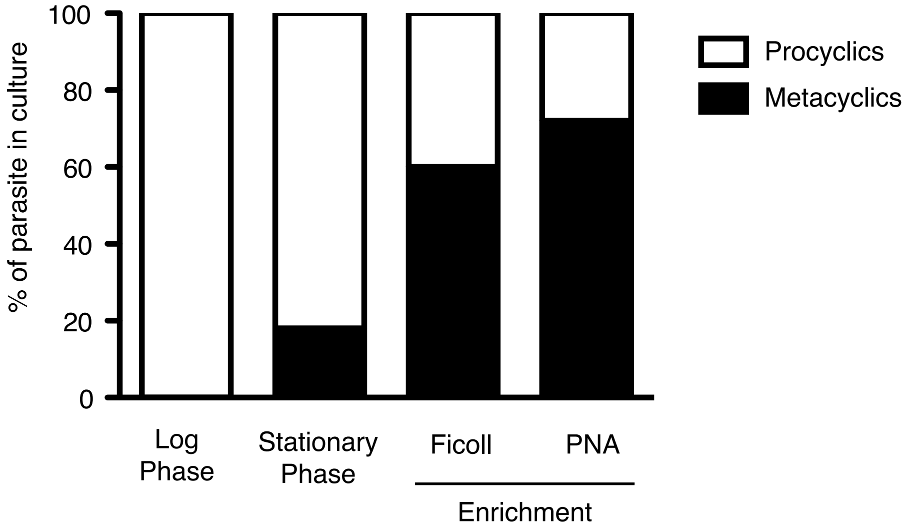

C

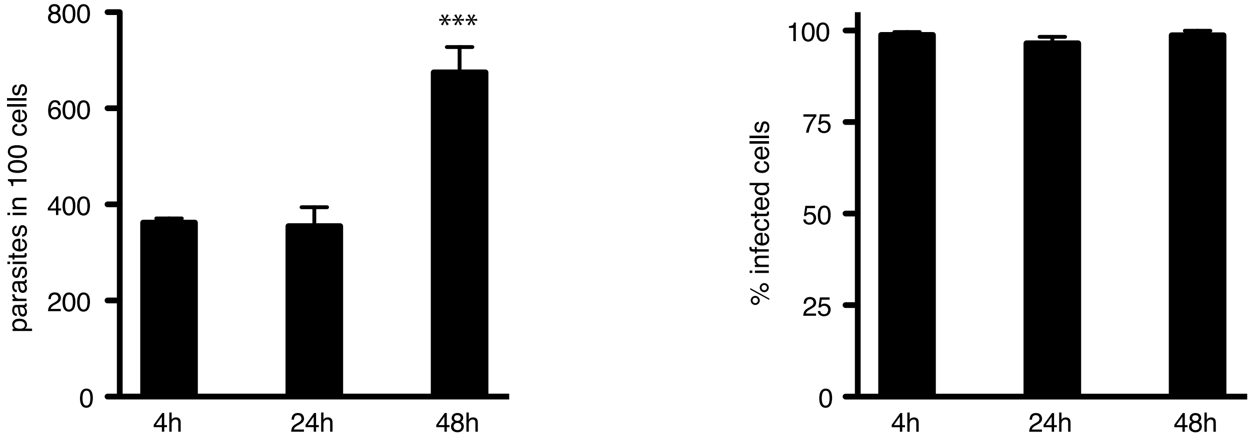

Figure S4

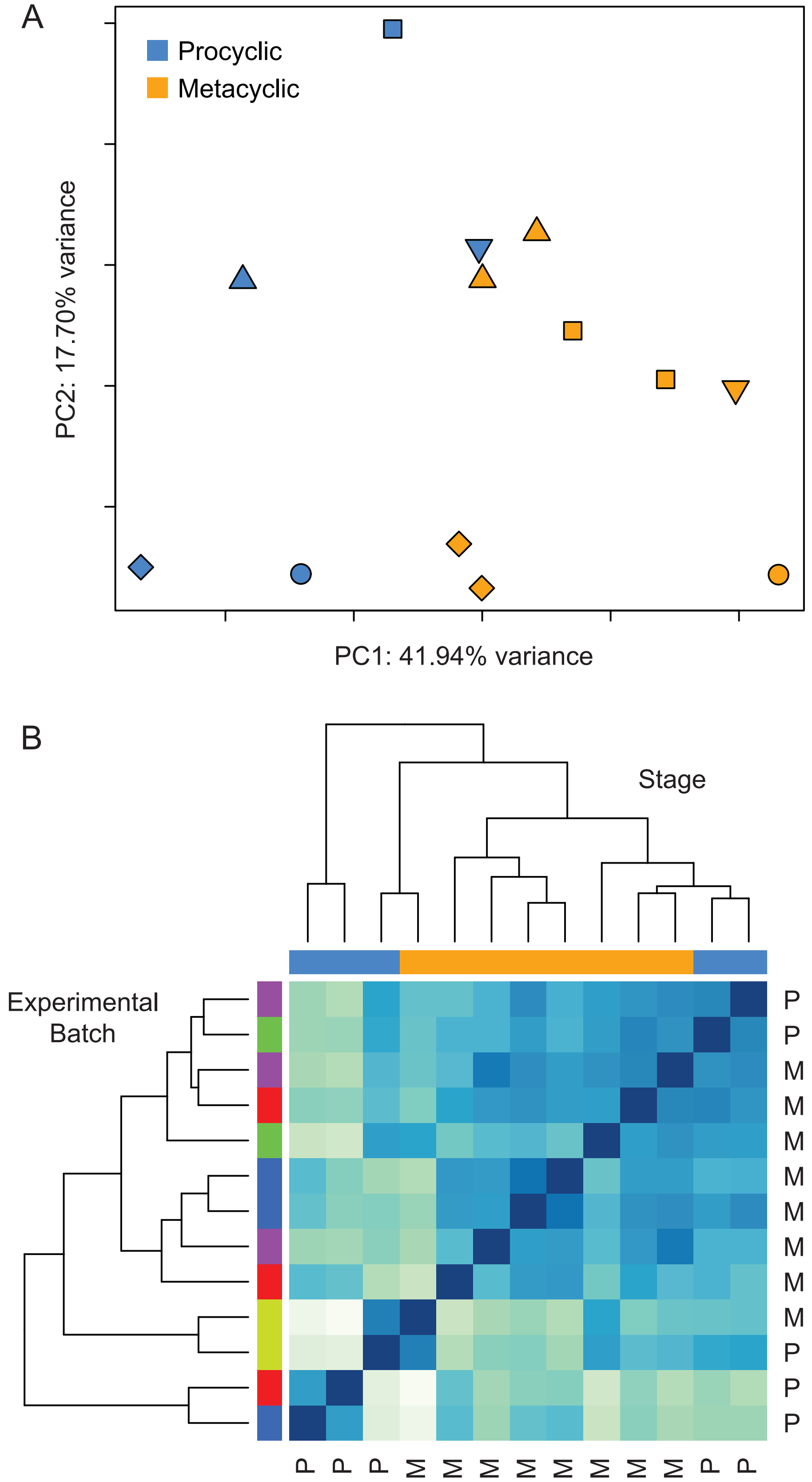
A
Procyclic
Metacyclic
PC2: 17.70% variance
PC1: 41.94% variance
B
Stage
Experimental Batch
P
P
M
M
M
M
M
M
M
M
P
P
P
P P P M M M M M M M M P P

Figure S5A

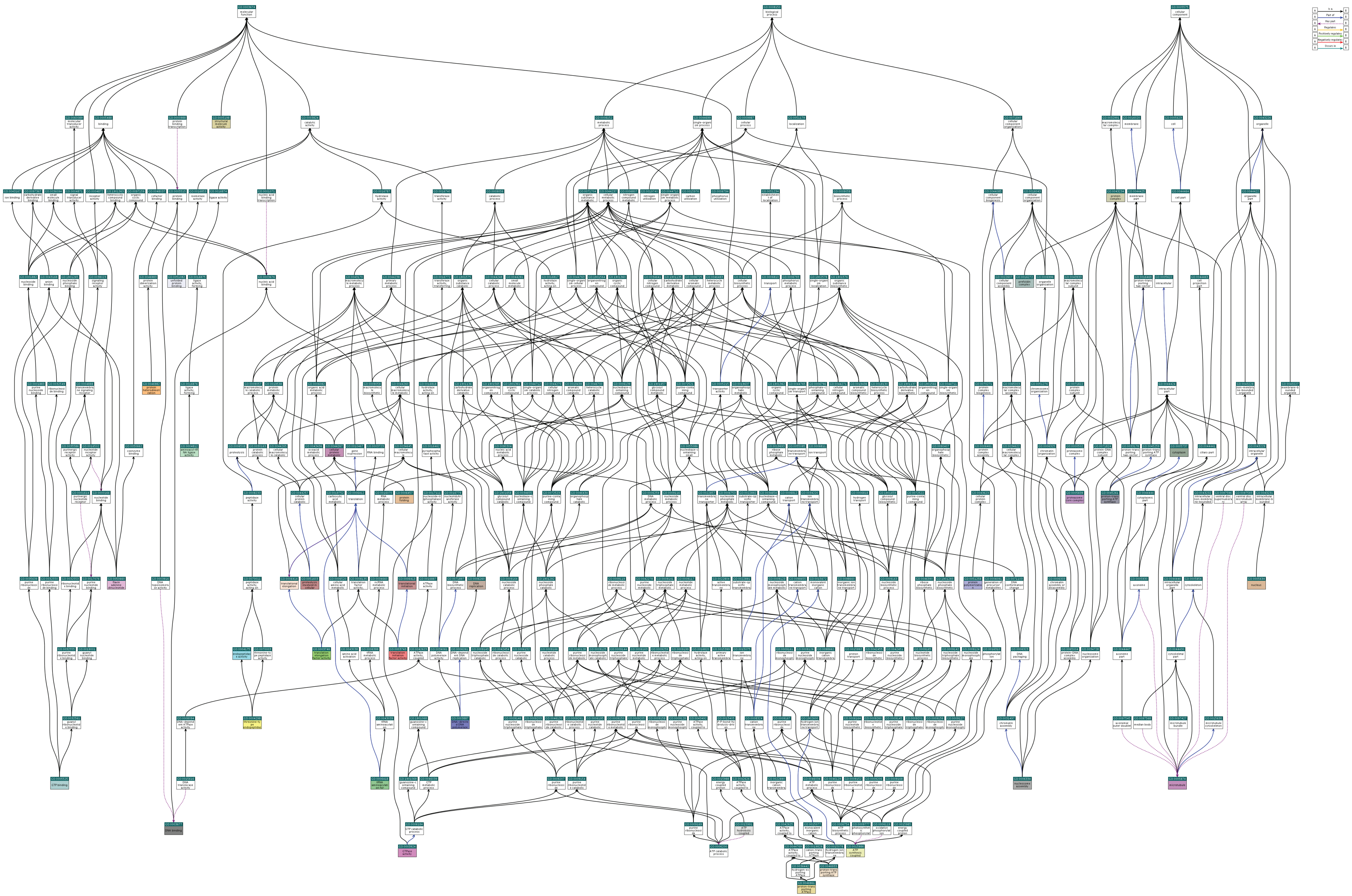

QuickGO - http://www.ebi.ac.uk/QuickGO

Figure S5B

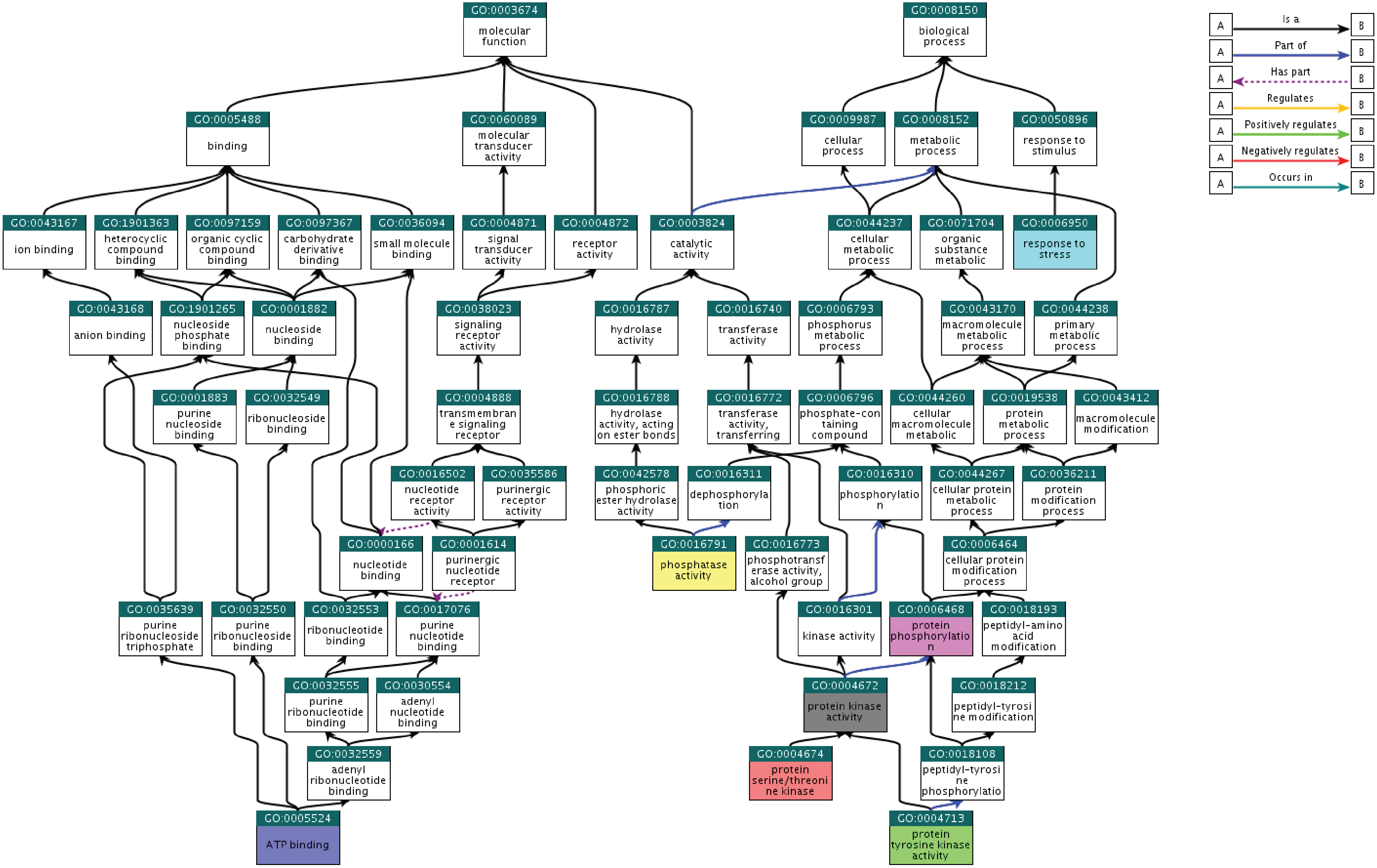

QuickGO - http://www.ebi.ac.uk/QuickGO

Figure S6

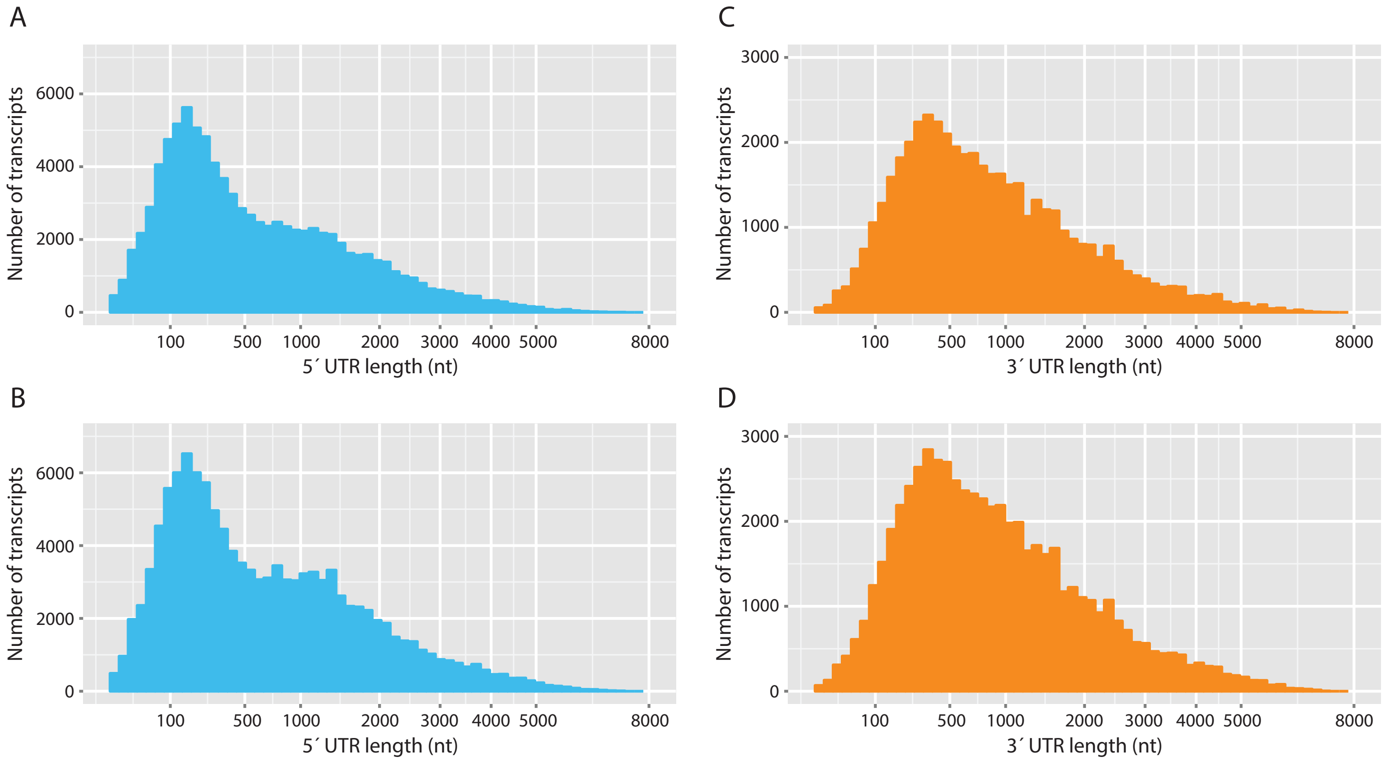
A
B
C
D
Number of transcripts
5´ UTR length (nt)
3´ UTR length (nt)
0
2000
4000
6000
1000
3000
100
500
1000
2000
3000
4000
5000
8000

Figure S7

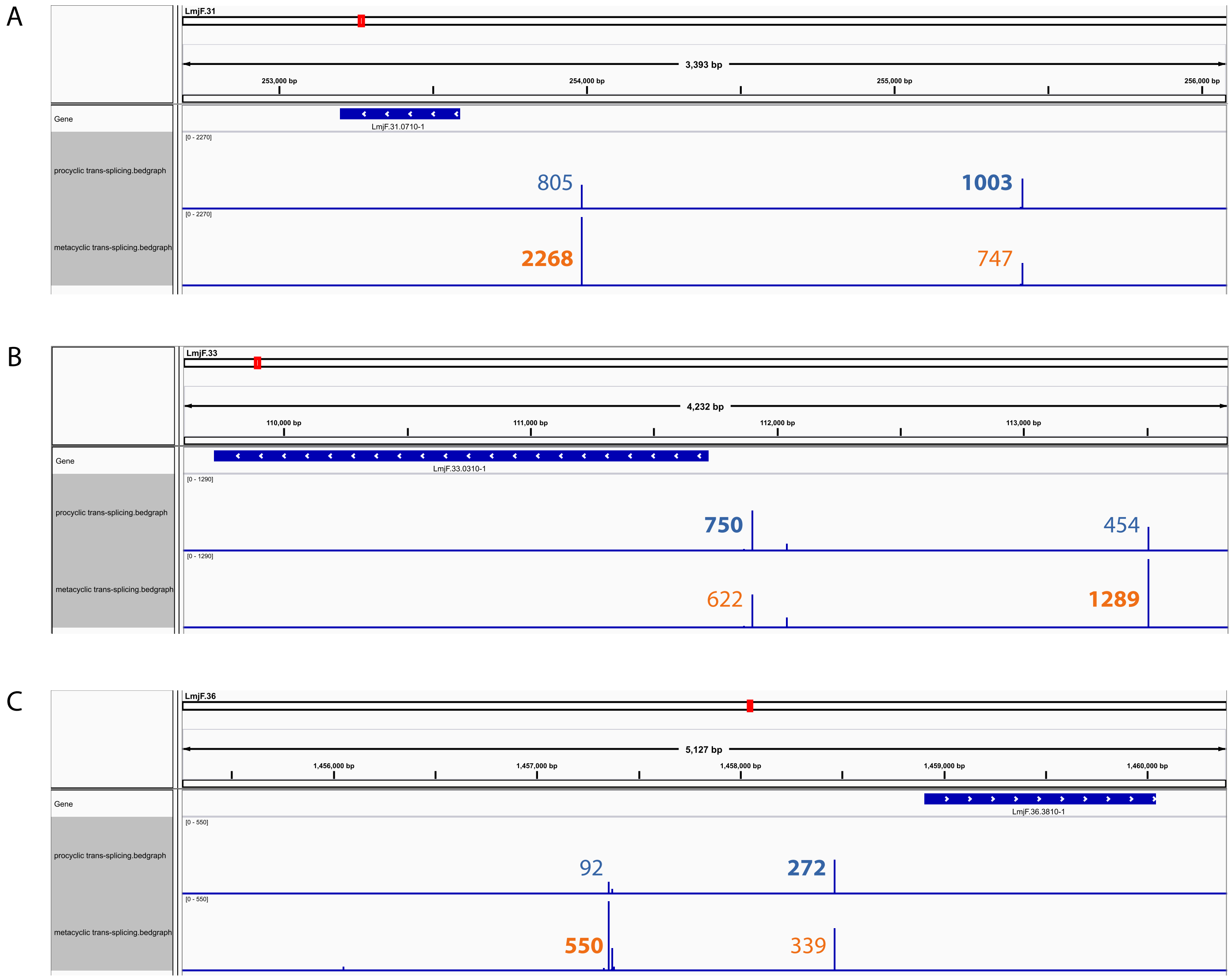

A
LmjF.31
3,393 bp
253,000 bp
254,000 bp
255,000 bp
256,000 bp
Gene
LmjF.31.0710-1
procyclic trans-splicing.bedgraph
[0 - 2270]
805
1003
metacyclic trans-splicing.bedgraph
[0 - 2270]
2268
747
B
LmjF.33
4,232 bp
110,000 bp
111,000 bp
112,000 bp
113,000 bp
Gene
LmjF.33.0310-1
procyclic trans-splicing.bedgraph
[0 - 1290]
750
454
metacyclic trans-splicing.bedgraph
[0 - 1290]
622
1289
C
LmjF.36
5,127 bp
1,456,000 bp
1,457,000 bp
1,458,000 bp
1,459,000 bp
1,460,000 bp
Gene
LmjF.36.3810-1
procyclic trans-splicing.bedgraph
[0 - 550]
92
272
metacyclic trans-splicing.bedgraph
[0 - 550]
550
339

Table S1. Experimental Design

ID for this Manuscript	Sample ID	SRA Accession Number	Promastigote Stage (Enrichment Method)	Batch	Number of reads passing Illumina filter	Number of reads mapped	Percentage of reads mapped
1	HPGL0075	n/a	procyclic	A	62,051,890	55,352,314	89.20
2	HPGL0076	n/a	metacyclic (Ficoll)	A	52,660,754	47,712,265	90.60
3	HPGL0096	SRR1460763	procyclic	B	103,466,044	92,748,560	89.64
4	HPGL0097	SRR1460764	metacyclic (PNA)	B	76,253,690	69,557,423	91.22
5	HPGL0098	SRR1460765	metacyclic (Ficoll)	B	93,319,752	84,580,995	90.64
6	HPGL0164	SRR1460766	procyclic	C	46,155,070	42,801,506	92.73
7	HPGL0165	SRR1460767	metacyclic (Ficoll)	C	45,492,872	41,811,082	91.91
8	HPGL0192	SRR1460768	procyclic	D	64,505,484	58,857,640	91.24
9	HPGL0193	SRR1460769	metacyclic (Ficoll)	D	70,178,176	63,898,517	91.05
10	HPGL0228	SRR1460770	procyclic	E	105,948,882	98,120,201	92.61
11	HPGL0229	SRR1460771	metacyclic (PNA)	E	77,161,294	71,310,256	92.42
12	HPGL0230	SRR1460772	metacyclic (Ficoll)	E	84,056,646	77,364,378	92.04
13	HPGL0324	SRR1460773	procyclic	F	69,215,652	63,766,203	92.13
14	HPGL0325	SRR1460774	metacyclic (PNA)	F	64,195,828	59,048,832	91.98
15	HPGL0326	SRR1460775	metacyclic (Ficoll)	F	60,167,474	55,318,601	91.94
Total					1,074,829,508	982,248,773	91.39

ID for this Manuscript	Number of SL-containing reads	Percentage of reads containing SL sequence	Number of polyA-containing reads	Percentage of reads containing polyA sequence
1	n/a	n/a	n/a	n/a
2	n/a	n/a	n/a	n/a
3	4,095,395	3.96	58,064	0.06
4	2,658,344	3.49	56,582	0.07
5	3,634,321	3.89	47,581	0.05
6	1,772,321	3.84	39,718	0.09
7	1,360,561	2.99	44,785	0.10
8	2,359,763	3.66	43,034	0.07
9	2,439,369	3.48	48,044	0.07
10	3,252,367	3.07	49,416	0.05
11	2,645,944	3.43	41,414	0.05
12	3,034,977	3.61	41,102	0.05
13	3,752,380	5.42	13,684	0.02
14	3,355,270	5.23	13,606	0.02
15	3,064,443	5.09	12,270	0.02
Total	37,425,455	3.90	509,300	0.05

Table S2. Dinucleotide acceptor site usage frequency

Acceptor Sequence	Primary	Minor
AG	96.80%	42.81%
TG	0.68%	11.27%
GG	0.56%	9.35%
CG	0.47%	7.79%
TT	0.37%	3.35%
GC	0.21%	3.41%
GT	0.14%	3.31%
TC	0.14%	2.16%
CA	0.11%	3.07%
AC	0.11%	2.35%
AT	0.11%	2.32%
GA	0.08%	2.11%
CT	0.07%	2.07%
CC	0.06%	1.89%
AA	0.04%	1.56%
TA	0.04%	1.19%

SUPPLEMENTARY FIGURE LEGENDS

Figure S1. Sample diagnostics to globally assess data similarities and identify outliers. RNA-seq was carried out using the Illumina platform on *L. major* procyclic and metacyclic promastigotes. Letters (A-F) in the sample name refer to experimental batch. Numbers are unique identifiers as shown in Table S1. Samples identified as outliers are indicated with an asterisk. A.) *Distribution of normalized gene counts by sample.* For each sample, counts were normalized for sequencing library size and a box plot was generated to compare the distribution of per-gene counts (log2 counts per million with an offset of 1). The ends of the whiskers represent the lowest datum still within 1.5 interquartile range (IQR) of the lower quartile, and the highest datum still within 1.5 IQR of the upper quartile. Gene features with extremely high or low expression levels are shown as open circles above and below the whiskers, respectively. B.) *Heatmap of Pearson correlation between samples.* Raw count data were used to generate a heatmap to illustrate the Pearson correlation between samples. The color key and histogram for the frequency of correlation values (range of 0.85-1) is shown below the heatmap. C.) *Median pairwise correlation.* Raw count data were used to compute the median pairwise correlation between each sample and all other samples. The median pairwise correlation across all samples was used to establish a cutoff value to identify outlier samples (dotted line). Samples are colored according to stage (blue=procyclic, orange=metacyclic).

Figure S2. Mean-variance curve modeling and fitting of a local regression trend line by `voom`. After log-transforming the quantile-normalized data, the `voom` function in `limma` was used to compute the mean-variance relationship for the transformed data and to generate gene weights that are used for the subsequent differential expression analysis. The relationship between mean expression (log2 counts per million with an offset of 0.5) and variance were modeled by `voom` and a trend line was created using a local regression (loess). Trend line values (red line) are robust to genes with high variability and are used as gene weights by `limma`.

Figure S3. Sample characterization. A. Phase contrast images of log-phase procyclic promastigotes, stationary phase promastigotes prior to enrichment for metacyclics, promastigotes following negative selection by PNA, and Ficoll-purified promastigotes. The bar in each panel represents 5 μm. **B.** Relative percentages of procyclic and metacyclic promastigotes in culture prior to and after the application of enrichment methods, as determined by counting 15 fields. **C.** Infections were established in peritoneal macrophages isolated from C57BL/6 mice using Ficoll-purified metacyclic promastigotes at an MOI of 5:1 in the presence of C5-deficient serum from DBA mice. Plots show the number of parasites observed per macrophage and the percentage of infected macrophages observed over the first 48 hours of the infection from one representative experiment. Asterisks indicate a significant difference in the number of parasites per 100 cells at 48 hours.

Figure S4. Principal Component Analysis (PCA) and hierarchical clustering analysis before accounting for batch effects. RNA-seq was carried out on *L. major* procyclic (log phase) promastigotes and metacyclic promastigotes isolated after enrichment using Ficoll or PNA. A principal component analysis (PCA) plot (A) and heatmap of a hierarchical clustering analysis using the Euclidean distance metric (B) are shown. Both analyses were performed on all *L. major* annotated genes (8,475) after filtering for low counts and quantile normalization. In the PCA plot, each point represents an experimental sample with point color indicating *L. major* developmental stage (blue = procyclic promastigote, orange = metacyclic promastigote) and point shape indicating batch/experimental date. Colors along the top of the heatmap indicate the developmental stage (blue = procyclic promastigote, orange = metacyclic promastigote) and colors along the left side of the heatmap indicate the batch/experimental date.

Figure S5. Gene ontology trees. Enriched GO terms were visualized as an ancestor chart using QuickGO (1) for genes (A) downregulated and (B) upregulated in metacyclic promastigotes relative to procyclic promastigotes. Colored boxes show GO terms included in the input dataset.

Figure S6. UTR length distribution by developmental stage. *Trans*-splicing sites were identified for each developmental stage and used to determine the coordinates and lengths of 5´ UTRs for procyclic promastigotes (**A**) and metacyclic promastigotes (**B**). An analysis of polyadenylation sites in each developmental stage was performed to determine 3´ UTR coordinates and lengths for procyclic promastigotes (**C**) and metacyclic promastigotes (**D**).

Figure S7. Visualization of changes in primary *trans*-splicing sites across developmental stages. The Integrative Genomics Viewer (IGV) (2) was used to visualize the number of reads that mapped to each *trans*-splicing site for three genes which showed a change in the preferred primary site between developmental stages and had a significant number of reads mapped to each primary site - LmjF.31.0710 (**A**), LmjF.33.0310 (**B**), and LmjF.36.3810 (**C**). The number of reads that mapped to each primary site (bold) and secondary site are shown for both procyclic promastigotes (blue text) and metacyclic promastigotes (orange text).

Table S1. Experimental design. Samples are listed using an internal lab sample identifier (HPGL----), which is referenced in the record stored at the Short Read Archive (accession numbers provided), and an additional simple identifier (1-15) for clarity in the text and figures. Experimental batches (A-F) are defined based on the start date of the experiment with each batch originating from a separate growth of cells. The number of reads sequenced, number and percentage of reads mapping to the *L. major* genome (v. 6.0), number and percentage of reads containing evidence of the SL sequence, and number and percentage of reads containing evidence of polyadenylation are also included.

Table S2. Dinucleotide acceptor site usage frequency. The percentage of primary and minor *trans*-splicing sites that use each dinucleotide acceptor sequence.

Dataset S1. Coordinates of novel ORFs. Novel open reading frames of at least 90 nucleotides in length were identified by manual annotation of translational evidence from a ribosome profiling study of *L. major* procyclic promastigote samples.

Dataset S2. Results from differential expression analysis of *L. major* metacyclogenesis using `limma` (protein-coding genes from TriTrypDB v 6.0). A. Significant genes (*P* values < 0.05). **B.** All genes (no *P* value cutoff applied).

Dataset S3. Results from differential expression analysis of *L. major* metacyclogenesis using `limma` (protein-coding genes from TriTrypDB v 6.0 and 1,044 novel ORFs). A. Significant genes (*P* values < 0.05). **B.** All genes (no *P* value cutoff applied).

Dataset S4. Enriched GO categories and corresponding differentially expressed genes. GOseq was used to identify gene ontology (GO) categories enriched for genes downregulated or upregulated in metacyclic promastigotes relative to procyclic promastigotes. For each enriched GO category, the *P* value for the enrichment is reported, as are the number and identity of DE genes in the dataset and the total number of *L. major* genes in the GO category.

Dataset S5. UTR coordinates. The *trans*-splicing sites and start coordinate of each associated CDS/ORF were used to determine 5´ UTR coordinates. The CDS/ORF end coordinate and associated polyadenylation sites were used to determine 3´ UTR coordinates. For each UTR coordinate, the number of reads that mapped to that site in each developmental stage are reported.

SUPPLEMENTARY REFERENCES

1. Binns, D., Dimmer, E., Huntley, R., Barrell, D., O'Donovan, C. and Apweiler, R. (2009) QuickGO: a web-based tool for Gene Ontology searching. *Bioinformatics,* **25**, 3045-3046.
2. Robinson, J.T., Thorvaldsdóttir, H., Winckler, W., Guttman, M., Lander, E.S., Getz, G. and Mesirov, J.P. (2011) Integrative genomics viewer. *Nat Biotechnol,* **29**, 24-26.
